# Gene expression profiling of brain endothelial cells after experimental subarachnoid haemorrhage

**DOI:** 10.1038/s41598-021-87301-z

**Published:** 2021-04-09

**Authors:** Michael K. Tso, Paul Turgeon, Bert Bosche, Charles K. Lee, Tian Nie, Josephine D’Abbondanza, Jinglu Ai, Philip A. Marsden, R. Loch Macdonald

**Affiliations:** 1 Division of Neurosurgery, University of Calgary, Calgary, AB Canada; 2 Division of Neurosurgery, St. Michael’s Hospital, Keenan Research Centre for Biomedical Science and the Li Ka Shing Knowledge Institute of St. Michael’s Hospital, University of Toronto, Toronto, ON Canada; 3 Division of Nephrology, University of Toronto, Toronto, ON Canada; 4 Department of Neurocritical Care, Neurological and Neurosurgical First Stage Rehabilitation and Weaning, MediClin Clinic Reichshof, Reichshof-Eckenhagen, Germany; 5 Institute of Neurophysiology, University of Cologne, Cologne, Germany; 6 Department of Neurology, University of Duisburg-Essen, Essen, Germany; 7 Department of Neurological Surgery, UCSF Fresno Campus, Fresno, USA

**Keywords:** Blood-brain barrier, Cerebrovascular disorders

## Abstract

Subarachnoid haemorrhage (SAH) is a type of hemorrhagic stroke that is associated with high morbidity and mortality. New effective treatments are needed to improve outcomes. The pathophysiology of SAH is complex and includes early brain injury and delayed cerebral ischemia, both of which are characterized by blood–brain barrier (BBB) impairment. We isolated brain endothelial cells (BECs) from mice subjected to SAH by injection of blood into the prechiasmatic cistern. We used gene expression profiling to identify 707 unique genes (2.8% of transcripts, 403 upregulated, 304 downregulated, 24,865 interrogated probe sets) that were significantly differentially expressed in mouse BECs after SAH. The pathway involving prostaglandin synthesis and regulation was significantly upregulated after SAH, including increased expression of the *Ptgs2* gene and its corresponding COX-2 protein. Celecoxib, a selective COX-2 inhibitor, limited upregulation of *Ptgs2* in BECs. In this study, we have defined the gene expression profiling of BECs after experimental SAH and provide further insight into BBB pathophysiology, which may be relevant to other neurological diseases such as traumatic brain injury, brain tumours, ischaemic stroke, multiple sclerosis, and neurodegenerative disorders.

## Introduction

Spontaneous subarachnoid haemorrhage (SAH) is a type of stroke that usually results from rupture of an intracranial aneurysm. Although it accounts for about 5% of all stroke, it is a particularly cruel affliction after which only about 25% of patients recover fully^1^. The etiology of brain injury after SAH encompasses factors such as transient global ischaemia, focal cerebral ischaemia, toxicity due to subarachnoid blood and secondary insults including direct brain damage from intracerebral haemorrhage, hydrocephalus, delayed cerebral ischaemia, and increased intracranial pressure^1,2^. Preclinical studies suggest a common thread underlying these aetiologies is damage to the brain microvasculature. Microvascular vasoconstriction, poor vasomotor propagation, microthrombi, increased leukocyte-endothelial cell interaction, inversion of neurovascular coupling, and blood–brain barrier (BBB) disruption all occur after experimental SAH^2,3^. More limited data in humans also supports the existence of these pathophysiologic processes after SAH^4–6^.

Targeting brain endothelial cells (BECs) of the BBB represents an ongoing therapeutic challenge^7^. Gene expression studies of BECs are lacking in SAH animal models. Our goal was to identify new therapeutic targets in SAH, specifically investigating brain endothelial factors in this hypothesis-generating study. We have isolated BECs from mice undergoing SAH using a prechiasmatic injection model and performed gene expression profiling. In this study, we identified the gene *Ptgs2* (prostaglandin-endoperoxide synthase 2) and its corresponding protein COX-2 (Cycooxygenase-2) as a potential treatment target in SAH.

## Results

### Experimental SAH caused neurobehavioural impairment and brain injury

**Figure 1:**
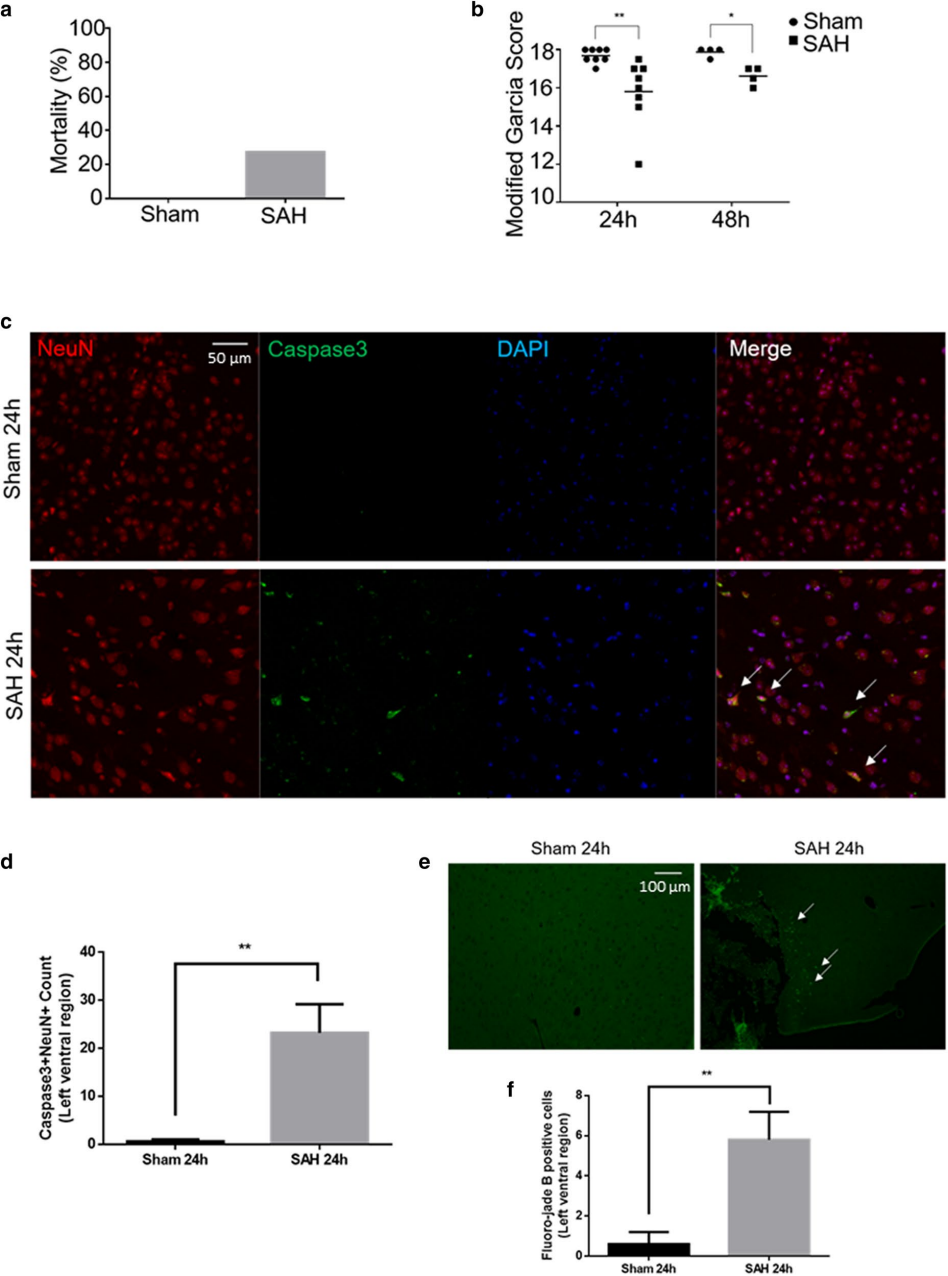
Experimental SAH neurobehavioural and histological assessments. (**a**) Mortality after SAH induction or sham procedure. (**b**) Modified Garcia Score at 24 h and 48 h after SAH induction or sham procedure (maximum normal score is 18). (**c**) Merged triple-stained immunofluorescent confocal microscopy of coronal brain slices 24 h after SAH induction or sham procedure (NeuN (neuronal marker) = red, Caspase-3 (apoptosis marker) = green, DAPI (nuclei marker) = blue). Arrows point to apoptotic neurons. (**d**) Semi-automated apoptotic neuronal count from left ventral region of coronal brain slices. (**e**) Fluorescent confocal microscopy with fluoro-jade B staining of coronal brain slices 24 h after SAH induction or sham procedure. Arrows point to degenerated neurons. (**f**) Semi-automated degenerated neuronal count from left ventral region of coronal brain slices. *n* = 8–11 per group for 1a-b. *n* = 4–5 per group for 1c-f. Data presented as means ± SEM (Standard error of the mean). Kruskal–Wallis test (Modified Garcia Score). *t*-test (Caspase 3, fluoro-jade B). *p < 0.05, **p < 0.01.

We had 4 experimental groups: SAH 24 h, Sham 24 h, SAH 48 h, and Sham 48 h (n = 8–11 per group). In the prechiasmatic blood injection mouse model of SAH, cerebral blood flow (CBF) acutely decreased after SAH with gradual recovery to 70–80% of baseline after 10 min (Supplementary Fig. S1). Mortality was 27% (3/11), typically occurring at the time of SAH (Fig. 1a). There was no mortality after a sham procedure, which involved insertion of a needle to the anterior cranial base without blood injection (0/8, Fig. 1a). No significant change in weight was seen after SAH (Supplementary Fig. S1). SAH caused neurobehavioural deficits based on the Modified Garcia Score at 24 h and 48 h (Fig. 1b, Supplementary Fig. S1). SAH caused histological evidence of brain injury, including neuronal apoptosis (Caspase3 + NeuN + cells) and neuronal degeneration (Fluoro-jade B + cells) (Fig. 1c–f).

### Experimental SAH caused blood–brain barrier impairment

**Figure 2:**
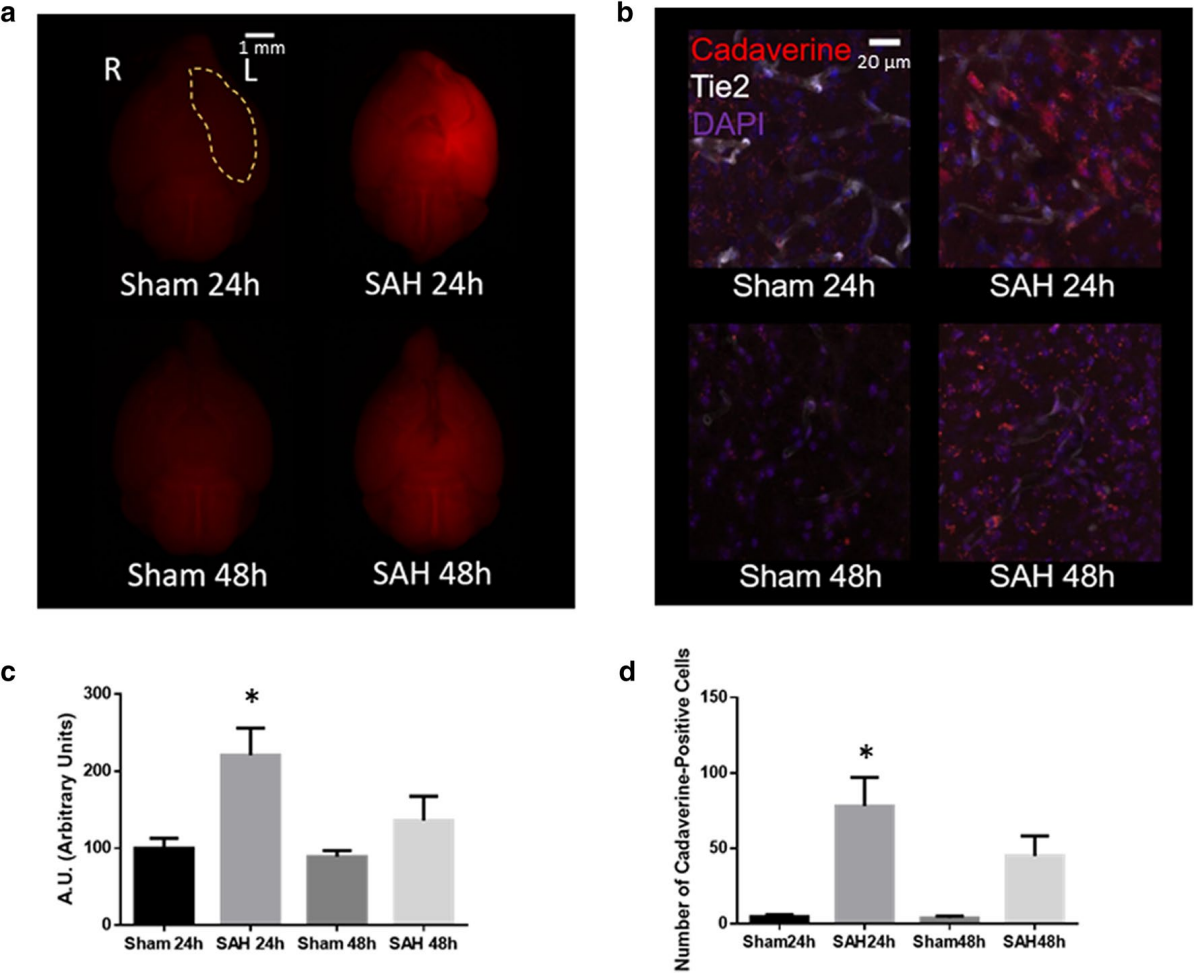
Blood–brain barrier (BBB) assessment after experimental SAH. (**a**) Whole brain fluorescent imaging from ventral perspective 24 h and 48 h after SAH or sham procedure. Left ventral region demarcated by yellow marking. Cadaverine dye fluorescence shown in red. (**b**) Confocal microscopy of coronal brain slices 24 h and 48 h after SAH or sham procedure. Cadaverine dye shown in red. Blood vessels shown in white with intrinsic Tie2-GFP fluorescence. Nuclei shown in blue with DAPI staining. (**c**) Quantification of cadaverine dye fluorescence from the left ventral region demarcated in (**a**). (**d**) Quantification of number of extravascular cadaverine-positive parenchymal cells. *n* = 3 per group. Data presented as means ± SEM. One-way ANOVA with Holm-Sidak *post-hoc* correction, *p < 0.05 (compared to Sham 24 h or Sham 48 h). Abbreviations: GFP = Green fluorescent protein. L = left. R = right.

In the prechiasmatic blood injection mouse model of SAH, BBB impairment was more prominent at 24 h than at 48 h as evidenced by extravascular leakage of fluorescent cadaverine dye (Fig. 2a,c). The leakage of fluorescent dye was particularly prominent in the left ventral cortex, in the region of the needle insertion, where the cadaverine dye was clearly extravascular with uptake into parenchymal cells (Fig. 2b,d). Consistent systemic intravascular uptake of the intraperitoneal-injected dye was seen, as evidenced by fluorescence of kidney tissue.

### Viable endothelial cells were isolated from mouse brains

**Figure 3:**
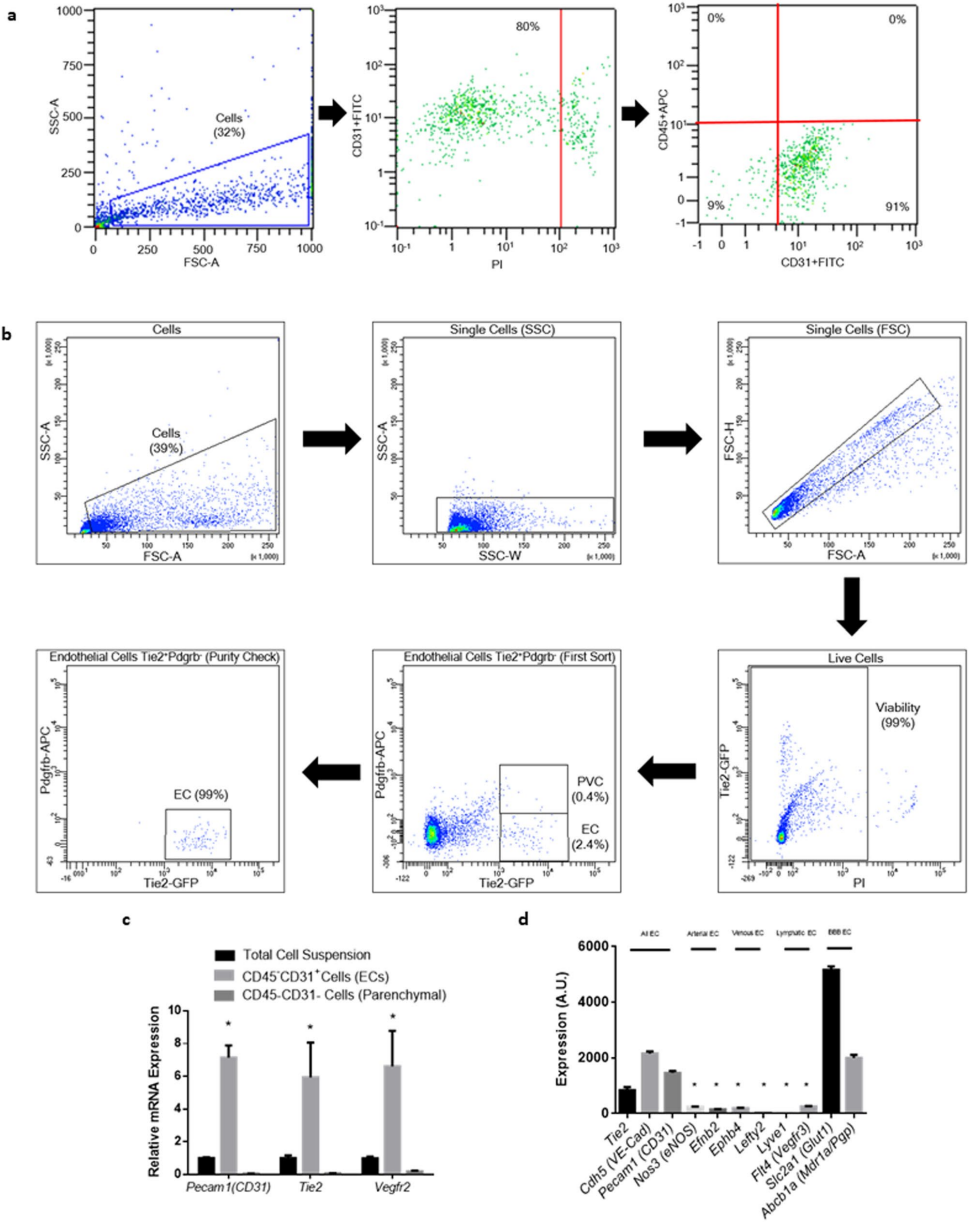
Isolation of brain endothelial cells (BECs). (**a**) Isolation of viable CD45–CD31 + endothelial cells by magnetic-beading sorting and confirmed by flow cytometry. (**b**) Isolation of viable Tie2 + Pdgfrb- endothelial cells by fluorescence-activated cell sorting (FACS). (**c**) Confirmation of CD45–CD31 + endothelial cell isolation by significant enrichment of endothelial mRNA transcripts relative to total cell suspension and CD45–CD31- brain parenchymal cells. *n* = 4 per group. Data presented as means ± SEM. One-way ANOVA with Holm-Sidak *post-hoc* correction, *p < 0.05. (**d**) Confirmation of predominantly blood–brain barrier (BBB) CD45–CD31 + endothelial cells by significant enrichment of BBB-associated genes measured by microarray intensities. *n* = 4 per group. Data presented as means ± SEM. One-way ANOVA with Holm-Sidak *post-hoc* correction. *p < 0.05 vs. “All EC” genes or “BBB EC” genes. Abbreviations: *Abcb1a*/Pgp/Mdr1a: ATP-binding cassette, sub-family B (MDR/TAP), member 1A/P-glycoprotein/Multidrug resistance protein 1a; APC: Allophycocyanin; *Cdh5*/VE-Cad: Cadherin 5; EC: Endothelial cell; *Efnb2*: Ephrin-B2; *Ephb4*: Ephrin type-B receptor 4; FITC: Fluorescein isothiocyanate; *Flt4*/*Vegfr3*: Fms-related tyrosine kinase 4/Vascular endothelial growth factor receptor 3; FSC-A: Forward scatter area; FSC-H = Forward scatter height; GFP: Green fluorescent protein; *Lefty2*: Left–right determination factor 2; *Lyve1*: Lymphatic vessel endothelial hyaluronan receptor 1; *Nos3*/eNOS: Nitric oxide synthase 3/endothelial nitric oxide synthase; *Pdgfrb*: Platelet-derived growth factor receptor beta; *Pecam1*: Platelet-endothelial cell adhesion molecule 1; PI: Propidium iodide; PVC: Perivascular cell; SSC-A: Side scatter area; *Slc2a1*/Glut-1: Solute carrier family 2, member 1/Glucose transporter 1; SSC-W: Side scatter width; *Tie2*: Tunica intima endothelial kinase 2; *Vegfr2*: Vascular endothelial growth factor receptor 2.

Endothelial cells were isolated from freshly extracted left cerebral hemispheres 24 h after SAH using magnetic-based methods (CD45–CD31 + cells). For confirmation of RT-PCR findings of CD45–CD31 + cells, endothelial cells were also isolated via fluorescence-activated cell sorting (FACS) (Tie2 + Pdgfrb- cells). These BEC suspensions had greater than 90% purity and greater than 80% viability (Fig. 3a–b, Supplementary Fig. S2). Each single biological replicate produced approximately 25,000 and 50,000 cells from the FACS-based and magnetic-based protocols, respectively. The isolated CD45–CD31 + endothelial cells revealed significant enrichment of endothelial gene transcripts (*Pecam1*, *Tie2*, *Vegfr2*) (Fig. 3c).

### RNA was successfully extracted and amplified from mouse brain endothelial cells

There was a relatively low amount of RNA extracted from CD45–CD31 + endothelial cells, so the RNA was amplified in order to perform gene expression profiling. This amplified RNA demonstrated enrichment of endothelial genes but relatively low levels of genes characteristic of other cell types (Supplementary Fig. S3). In addition, the amplified RNA was enriched in genes characteristic of endothelial cells derived from the BBB (Slc2a1/Glut1, Abcb1a/Mdr1a), but exhibited relatively low levels of genes characteristic of endothelial cells derived from arteries, veins, or lymphatic vessels (Fig. 3d). A fixed amount of exogenous mRNA plasmid was added to the endothelial cell suspension prior to RNA extraction, confirming similar first strand and amplification efficiencies between biological replicates (Supplementary Fig. S4). Gene expression patterns pre- and post-amplification were similar (Supplementary Fig. S4).

### SAH and sham mice demonstrated distinct endothelial gene expression patterns

**Figure 4:**
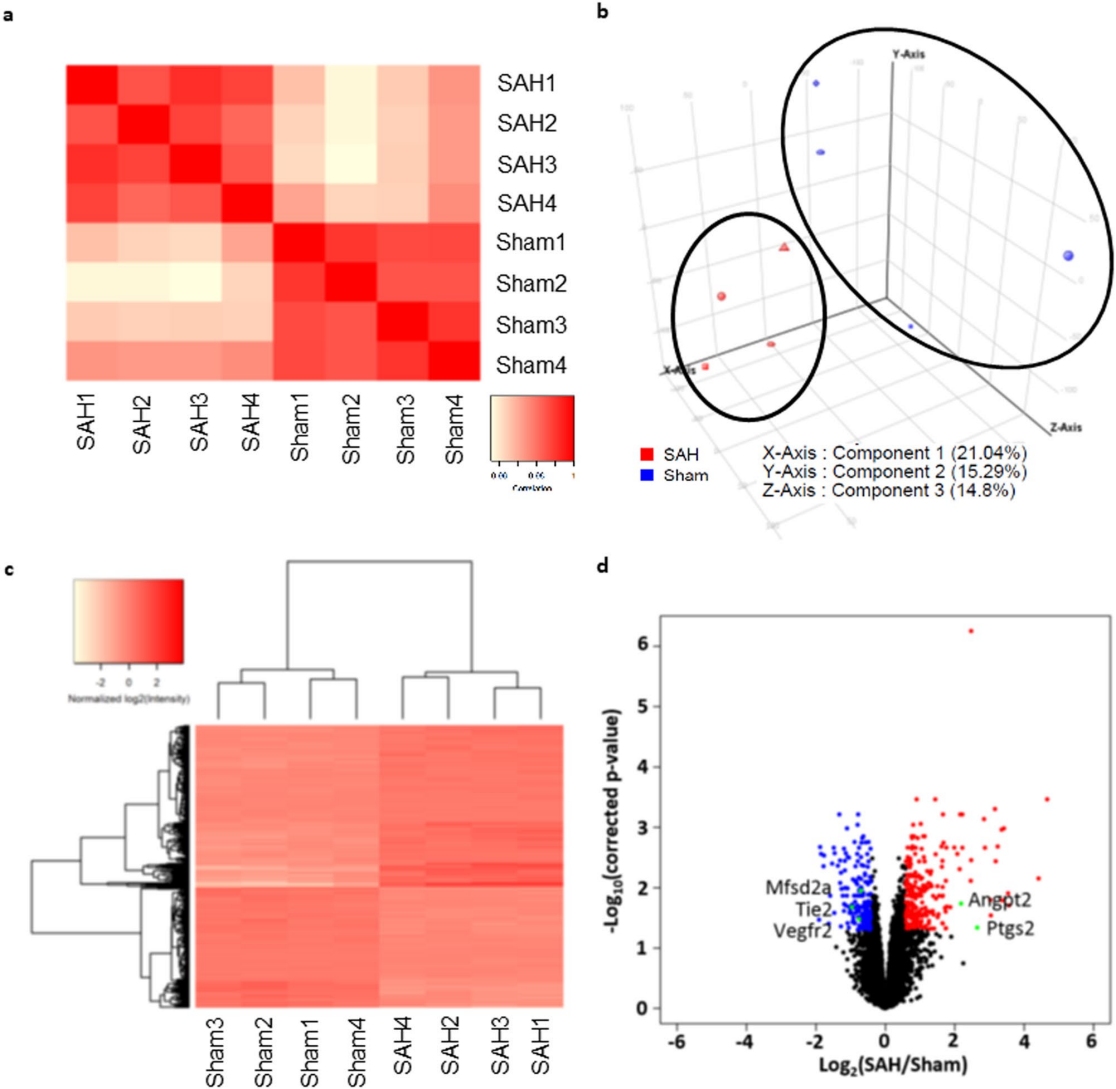
Gene expression profiling of CD45–CD31 + brain endothelial cells. (**a**–**c**) Pearson’s correlation, principal component analysis (PCA), and unsupervised hierarchical clustering revealed clustering of SAH and sham biological replicates based on gene expression profiling of CD45–CD31 + BECs. (**d**) Volcano plot demonstrating 707 genes with significant differential expression 24 h after SAH vs. sham procedure (2.8% of 24,865 interrogates probe sets). There were 403 upregulated genes and 304 downregulated genes. Red dots show the 200 genes that were significantly upregulated by more than 1.5-fold. Blue dots show the 304 genes that significantly downregulated to 75% of baseline. Specific genes of interest are labeled with green dots. *n* = 4 per group. Abbreviations: *Angpt2*: Angiopoietin 2; *Mfsd2a*: Major facilitator superfamily domain containing 2a; *Ptgs2*/COX-2: Prostaglandin-endoperoxide synthase 2; *Tie2*: Tunica intima endothelial kinase 2; *Vegfr2*: Vascular endothelial growth factor receptor 2.

**Figure 5:**
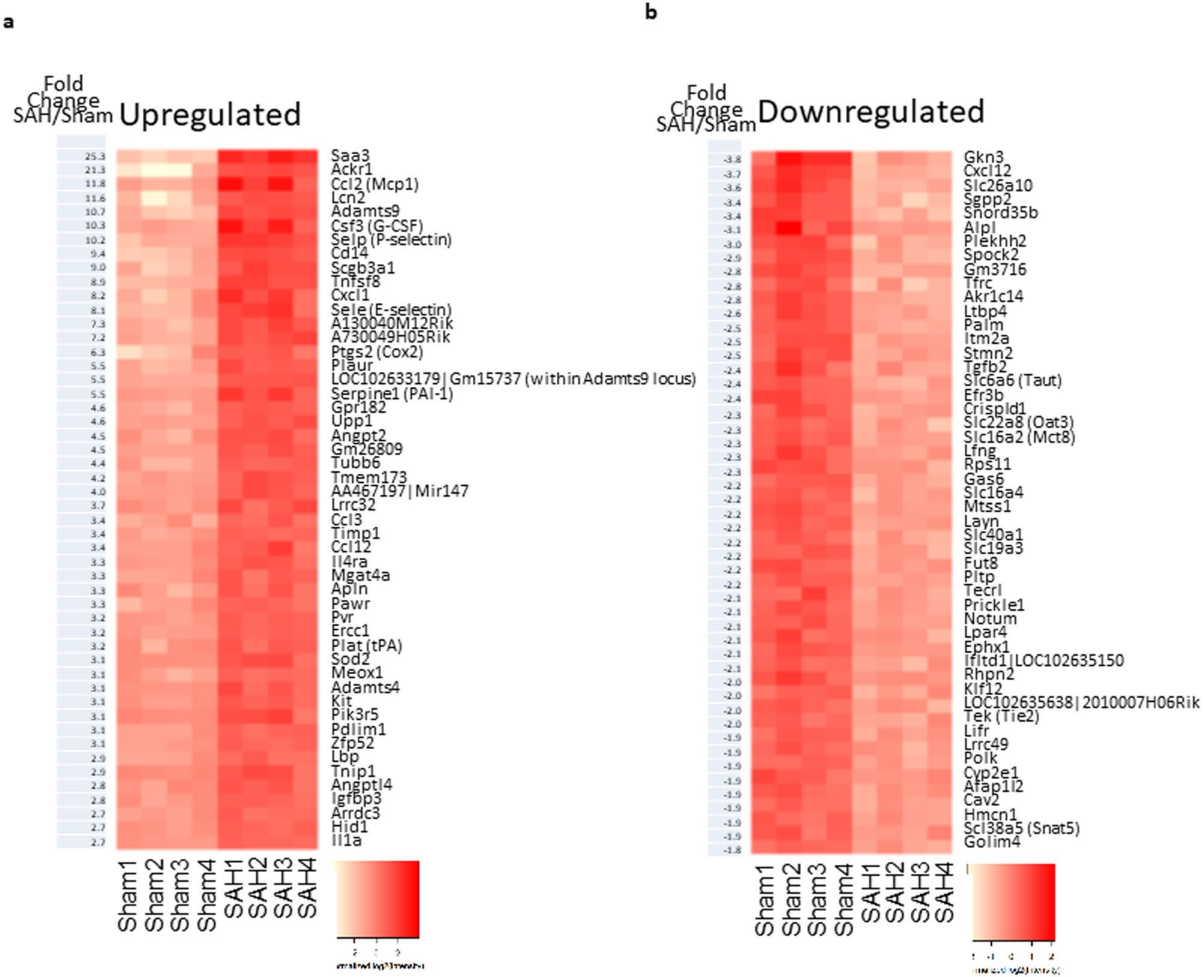
Differentially expressed genes. (**a**) The 50 highest ranked genes based on upregulation in CD45–CD31 + BECs after SAH. (**b**) The 50 highest ranked genes based on downregulation in CD45–CD31 + BECs after SAH.* n* = 4 per group.

Gene expression profiling of CD45–CD31 + BECs derived from SAH and sham mice showed distinct gene expression patterns based on Pearson’s correlation, principal component analysis (PCA), and unsupervised hierarchical clustering (Fig. 4a–c). Among 24,865 interrogated probe sets, 707 genes (2.8%) showed significant differential expression after SAH with 403 genes upregulated and 304 genes downregulated (Fig. 4d). In particular, 236 genes were significantly upregulated by at least 1.5-fold and 200 genes were significantly downregulated to less than 75% of baseline value. Genes with the highest fold changes are shown in Fig. 5a,b.

### Inflammatory response genes were significantly upregulated in endothelial cells after SAH

There were no significant changes in genes related to BBB intercellular junctions after SAH (Supplementary Fig. S5). There was an overall modest downregulation of genes related to BBB transporters after SAH (Supplementary Fig. S6). Gene set enrichment analysis (GSEA) was used to determine relevant biological processes and pathways affected after SAH. A number of gene ontology biological processes, cellular components, molecular functions and pathways were affected by SAH, including most prominently “Inflammatory Response” and “Prostaglandin Synthesis and Regulation” (Tables 1, 2, Supplementary Fig. S7).

**Table 1:** Gene set enrichment analysis (GSEA) with gene ontology (GO) data sets.

Gene ontology (GO) data sets	NES	FDR q-value
**GO—biological process**		
Inflammatory response	1.76	0.091
Heterophilic cell–cell adhesion	1.73	0.095
Oogenesis	1.73	0.087
Regulation of apoptotic process	1.72	0.081
Response to hydrogen peroxide	1.71	0.086
Positive regulation of camp biosynthetic process	1.71	0.084
Cell–cell adhesion	1.70	0.082
Peptide cross-linking	1.70	0.080
Cytokine production	1.69	0.084
Response to lipopolysaccharide	1.68	0.087
Activation of mapk activity	1.68	0.086
Placenta development	1.67	0.092
Regulation of blood pressure	1.67	0.089
Superoxide metabolic process	1.67	0.086
Response to oxidative stress	1.66	0.095
Adult locomotory behaviour	− 2.14	0.01
Synaptic transmission	− 1.95	0.024
**GO—cellular component**		
Secretory granule	1.73	0.081
High-density lipoprotein particle	1.71	0.081
**GO—molecular function**		
Calcium-dependent protein binding	1.79	0.093
Cytokine activity	1.75	0.097
Protease binding	1.70	0.083
Metallopeptidase activity	1.70	0.083
Transporter activity	1.69	0.077

*FDR* False-discovery rate, *NES* Normalized enrichment score.

**Table 2:** Gene set enrichment analysis (GSEA) with pathway analysis data sets.

Pathway analysis data sets	NES	FDR q-value
TH1 TH2 differentiation	1.81	0.042
Prostaglandin synthesis and regulation	1.75	0.067
Folate metabolism	1.70	0.071
Regulation of actin cytoskeleton	1.70	0.056
Selenium pathway	1.68	0.054
Monoamine transport	1.67	0.050
Inflammatory response pathway	1.66	0.051
EBV LMP1 signaling	1.65	0.056
Selective expression of chemokine receptors during T-cell polarization	1.64	0.072
Type II diabetes mellitus	1.64	0.076
Apoptosis	1.62	0.091
Complement and coagulation cascades	1.62	0.086
IL4 signaling pathway	1.61	0.093
Follicle stimulating hormone signaling pathway	1.61	0.088
Matrix metalloproteinases	1.60	0.089
IL-4 signaling pathway	1.60	0.086
Cytoskeletal regulation by rho gtpase	1.60	0.082
Role of mitochondria in apoptotic signaling	1.58	0.097
Caspase cascade in apoptosis	1.57	0.102
HIV-I NEF negative effector of FAS and TNF	1.56	0.098
Chaperones modulate interferon signaling pathway	1.55	0.099
KIT receptor signaling pathway	1.55	0.100
IL-2 receptor beta chain in T cell activation	1.54	0.099

*FDR* False-discovery rate, *NES* Normalized enrichment score.

### Experimental SAH led to increased Ptgs2 (Cox-2) and Angpt2 expression in brain endothelial cells

**Figure 6:**
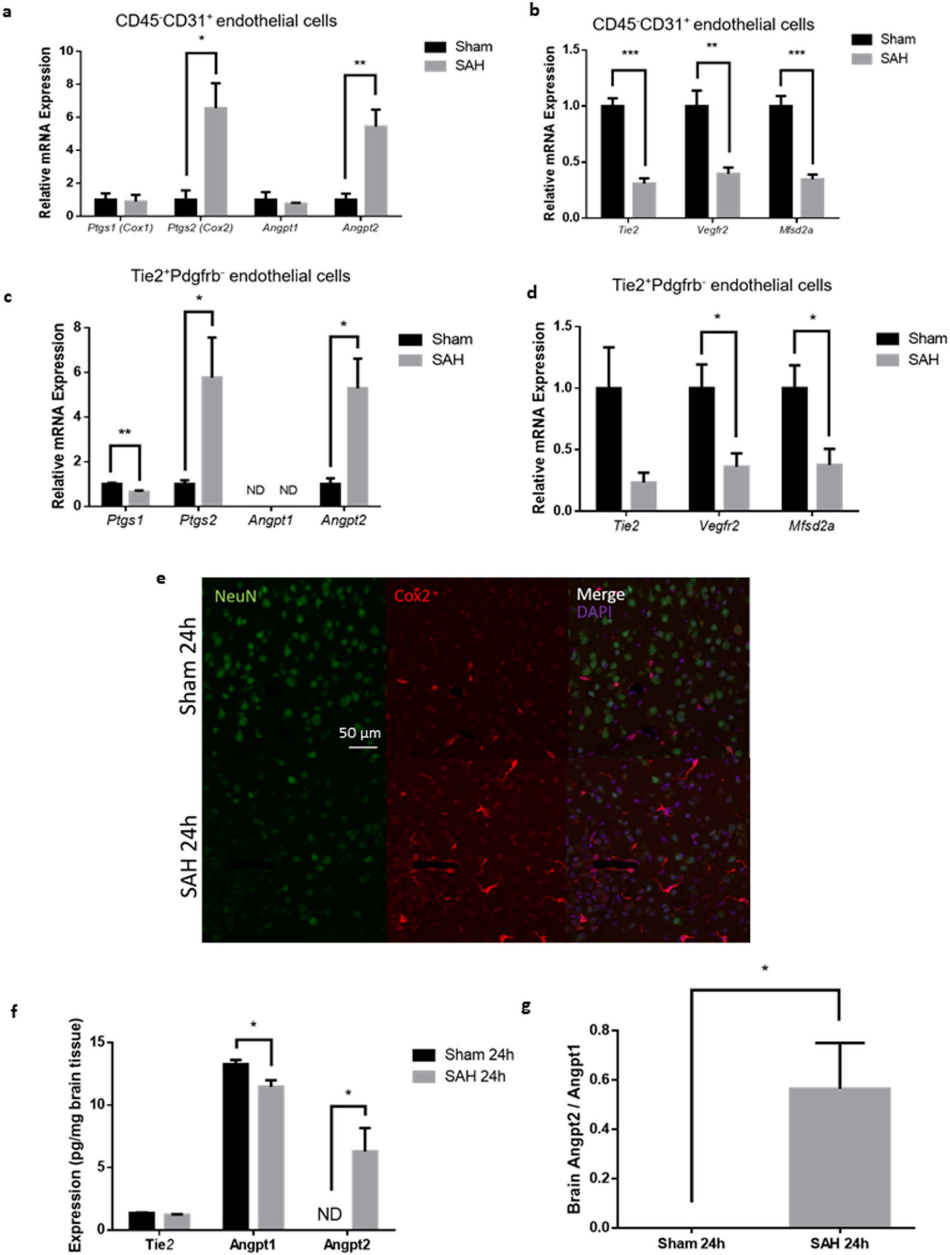
Expression of Ptgs2 and Angpt2 after experimental SAH. (**a**) Increased mRNA expression of *ptgs2* and *angpt2* in CD45–CD31 + BECs after SAH. (**b**) Decreased mRNA expression of *tie2*, *vegfr2*, and *mfsd2a* in CD45–CD31 + BECs after SAH. Increased mRNA expression of *ptgs2* and *angpt2* in Tie2 + Pdgfr- BECsafter SAH. (**b**) Decreased mRNA expression of *tie2*, *vegfr2*, and *mfsd2a* in Tie2 + Pdgfr-BECs after SAH. *n* = 4 per group. mRNA expression levels normalized to Actb (beta-actin). *t*-test with Holm-Sidak *post-hoc* correction *p < 0.05, **p < 0.01, ***p < 0.001. Data presented as means ± SEM. (**e**) Confocal microscopy images of coronal brain slices after SAH or sham procedure with neurons labeled with NeuN (green), Ptgs2 (Cox2) protein labelled in red, and nuclei labelled with DAPI (purple). *n* = 5 per group. (**f**) Protein expression of Tie2, Angpt1, and Angpt2 in left hemisphere brain homogenates after SAH or sham procedure using enzyme-linked immunosorbent assay (ELISA) kits. (**g**) Ratio of Angpt2 over Angpt1 in left hemisphere brain homogenates after SAH or shame procedure using ELISA kits. *n* = 5. *t*-test with Holm–Sidak *post-hoc* correction *p < 0.05. Abbreviations: *Angpt1/2*: Angiopoietin 1/2; *Cox1/2*: Cyclooxygenase 1/2; *Mfsd2a*: Major facilitator superfamily domain containing 2a; ND: Not detected; *Ptgs1/2*: Prostaglandin-endoperoxide synthase 1/2; *Vegfr2*: Vascular endothelial growth factor receptor 2.

Gene expression profiling of CD45–CD31 + BECs revealed increased expression of *ptgs2* (Prostaglandin-endoperoxide synthase 2, also known as Cyclooxygenase-2 or Cox-2) and *angpt2* (Angiopoetin-2) after SAH. These results were validated with real-time PCR and confirmed in Tie2 + Pdgfrb − BECs isolated by a completely different methodology (Fig. 6a–d). There was corresponding increased Cox-2 immunofluorescence in brain blood vessels and increased Angpt2 protein expression in brain tissue (Fig. 6e,f). Related genes Ptgs1 (Cox-1) and Angpt1 demonstrated no change in expression after SAH (Fig. 6a–d). After SAH, there was increased Angpt2/Angpt1 protein expression ratio in brain tissue but not in serum (Fig. 6g, Supplementary Fig. S8).

### Celecoxib limited upregulation of Ptgs2 (Cox-2) in brain endothelial cells after experimental SAH

**Figure 7:**
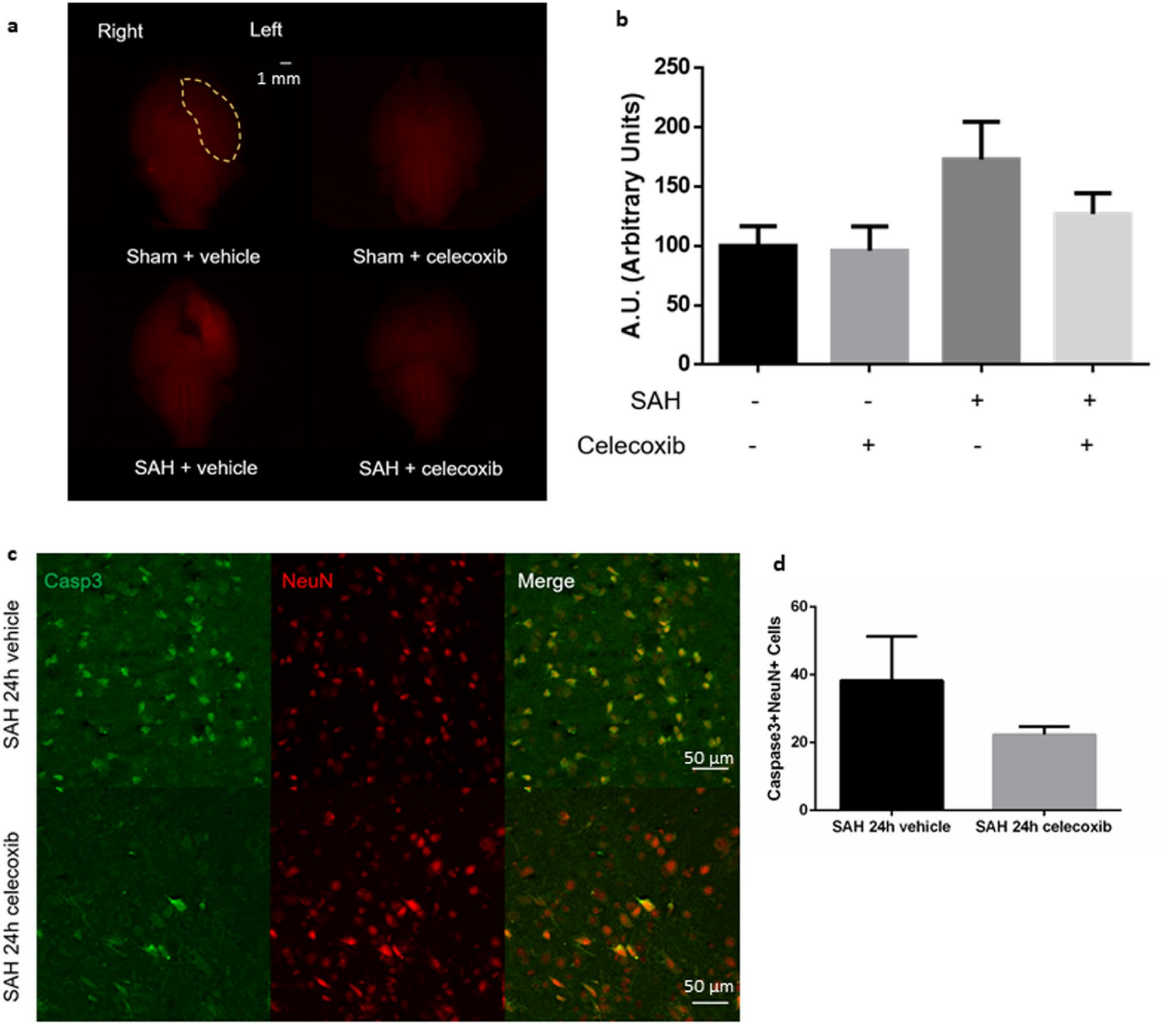
Celecoxib treatment after experimental SAH. (**a**) Whole brain fluorescent imaging from ventral perspective 24 h after SAH or sham procedure, treated with selective Cox2 inhibitor celecoxib or vehicle. Left ventral region demarcated by yellow marking. Cadaverine dye fluorescence shown in red. (**b**) Quantification of cadaverine dye fluorescence in left ventral region. (**c**) Merged double-stained immunofluorescent confocal microscopy of coronal brain slices 24 h after SAH treated with celecoxib or vehicle (NeuN (neuronal marker) = red, Caspase-3 (apoptosis marker) = green). Apoptotic neurons noted in yellow. (**d**) Semi-automated apoptotic neuronal count from left ventral region of coronal brain slices. *n* = 3–4 per group. Data presented as means ± SEM. One-way ANOVA with Holm-Sidak *post-hoc* correction. *p < 0.05.

The selective Cox-2 inhibitor celecoxib improved overall activity level but did not improve Modified Garcia Score after SAH (Supplementary Fig. S9). There was a trend toward decreased BBB disruption in the left ventral cortex in SAH mice treated with celecoxib (p = 0.15, Fig. 7a,b). However, there was no significant decrease in neuronal apoptosis/degeneration after SAH (Fig. 7c,d, Supplementary Fig. S9). Celecoxib did not affect Cox-2 protein expression in brain blood vessels but limited CD45–CD31 + BEC *ptgs2* gene upregulation after SAH (Supplementary Fig. S9).

## Discussion

Our study used a prechiasmatic injection model of SAH which demonstrated maximal BBB disruption at the 24 h time-point. We isolated BECs from mice with SAH. Using gene expression profiling with a microarray platform, we identified genes that were significantly differentially expressed in BECs after SAH. Pathways relevant to inflammation and prostaglandin signaling were upregulated in these cells. *Ptgs2* (Cox-2) was upregulated at both the RNA and protein level and may represent a potential therapeutic target in SAH.

Prior preclinical gene expression studies used combined whole brain tissue (neurons, oligodendrocytes, astrocytes, etc.) and brain blood vessels (endothelial cells, pericytes, vascular smooth muscle cell, etc.), which may obscure gene expression changes in specific cell types^8–14^. Tissue samples from patients with SAH are difficult to obtain and have been limited to whole blood, cerebrospinal fluid, aneurysm fundus, and endovascularly-obtained endothelial cells from aneurysm lumens^15–26^. However, such samples provide limited information regarding brain injury and the blood brain barrier, and hence the need for preclinical studies^7^. More in-depth studies investigating BEC gene expression patterns are needed, especially in models of neurological diseases including SAH^27^.

Our study revealed several biologically relevant targets in BECs after SAH (Fig. 5). There was significant upregulation of Ptgs2/COX-2 and we chose to investigate this target further due to overall upregulation of the prostaglandin synthesis pathways. COX-2, an inducible enzyme that generate various prostaglandins, has been well-studied and is druggable with the clinically available selective COX-2 inhibitor celecoxib. Unlike COX-1-derived prostaglandins which provide house-keeping functions and gastric cytoprotection, COX-2-derived prostaglandins are upregulated in states of inflammation and cancer in response to cytokines and mitogens^28^. It is not surprising that celecoxib did not affect the protein expression of COX-2 after SAH given that it is merely an inhibitor. Celecoxib may limit inflammation by limiting PGE2 production and signaling through pro-inflammatory EP2 receptors^29^. However, we were surprised that celecoxib downregulated the mRNA expression of Ptgs2/COX-2. The mechanism of this downregulation is unclear.

COX-2 is also known to be constitutively expressed in kidney and brain tissue^30^. It is known that celecoxib can cross the BBB^31^. Patients with SAH have been treated with COX-2 inhibitors without clear adverse reactions^32^. There is concern for increased pro-thrombotic complications including myocardial infarction and ischaemic stroke in the setting of prolonged use of COX-2 inhibitors in patients with vascular risk factors^33^. The VIGOR (Vioxx Gastrointestinal Outcomes Research) clinical trial comparing rofecoxib, a select COX-2 inhibitor and naproxen, a non-selective non-steroidal anti-inflammatory drug (NSAID) in patients with rheumatoid arthritis, found a significant increase in cardiovascular events in patients treated with rofecoxib^34^. The mechanism for the increased pro-thrombotic events was thought to be suppression of prostacyclin (PGI2), a vascular-protective prostaglandin^28^. For celecoxib, the PRECISION trial found no significant increased risk in myocardial infarction or stroke compared with ibuprofen or naproxen^35^. The lack of increased cardiovascular risk associated with celecoxib was also seen in large retrospective clinical studies^36,37^.

COX-2 inhibitors have not been studied extensively in SAH patients. In preclinical studies, NS398, a COX-2 inhibitor, was used as treatment in a mouse endovascular perforation SAH model and was found to be neuroprotective^38^. COX-2 has been shown to be involved in the development of vasospasm via increased expression of endothelin-1 and activation of the JAK-STAT signalling cascade (Janus kinase-Signal transducer and activator of transcription)^39–42^. Celecoxib treatment was shown to attenuate vasospasm in a preclinical SAH model^39^. Celecoxib is known to antagonize L-type calcium channels, which can cause vasodilation^43^. EP4 is a downstream receptor for PGE2, which is produced by COX-2, and its activation was found to provide neuroprotection and BBB stability, and its antagonism was found to provide the reverse effect in a rat endovascular perforation SAH model^44^. However, EP4 is only one of 4 receptors for PGE2 (EP1, EP2, EP3, EP4), with each receptor having context-dependent proinflammatory versus anti-inflammatory signaling^28^.

COX-2 inhibitors may have additional benefits in SAH, including sodium retention to counteract cerebral salt wasting, and increased blood pressure, which may be a beneficial treatment for delayed cerebral ischemia after the ruptured aneurysm has been secured. A retrospective analysis of the clinical trial CONSCIOUS-1 comparing clazosentan to placebo in patients with SAH found that NSAID treatment during hospital admission was associated with improved clinical outcomes and decreased mortality^32^. Unfortunately, only a small number of patients were administered COX-2 inhibitors, preventing any conclusions regarding specific NSAIDs.

Angiopoietins are vascular growth factors which include Angpt1, an important vascular maintenance regulator through its signalling via the Tie2 receptor, and Angpt2, a vascular disruptive factor due to its partial antagonism of Tie2 signalling^45^. In many inflammatory diseases, Angpt2 is upregulated^45^. Prior studies have shown that hypoxia may upregulate Angpt2 in a COX-2-dependent manner^46^. However, in our study, COX-2 inhibitors did not significantly attenuate upregulation of Angpt2 after SAH. There are likely alternative pathways that lead to Angpt2 upregulation aside from prostanoid signaling.

Our study has several limitations. Although the BEC isolation methods were optimized to provide maximal efficiency, we cannot exclude ex vivo effects on gene expression changes secondary to the isolation process itself. For example, ex vivo exposure to thrombin can upregulate COX-2 expression in endothelial cells^47^. Our study used BECs derived from the left cerebral hemisphere, which had maximal BBB disruption in our SAH model. There may, however, be heterogeneity of gene expression in BECs from different brain compartments. Our immunofluorescence experiments looked at Cox-2 protein expression in vascular structures but without specific colocalization with endothlelial markers. Therefore, it is possible that the increased Cox-2 protein expression may not be related to or exclusive to endothelial cells, but rather vascular smooth muscle cells, pericytes or perivascular macrophages. Although we have focused on endothelial-derived COX-2 signaling as a target after SAH, we have not excluded the potential beneficial effects of COX-2 antagonism in other cell types.

Future experiments could fully address the time-frame of BBB disruption in this prechiasmatic model of SAH including earlier and later time-points. Also, it would be interesting to see if temporal changes in BBB disruption correlate with endothelial Ptgs2/COX-2 expression. Further studies may examine the COX-2 downstream pathways including the expression and activity of mPGES-1, PGE2 and its respective receptors EP1-4.

In conclusion, we isolated BECs in an experimental SAH model and identified COX-2 as a potential therapeutic target. BBB impairment is part of the pathophysiology of several neurological diseases aside from SAH including ischaemic stroke, traumatic brain injury, multiple sclerosis, brain tumours, epilepsy, and neurodegenerative disorders such as Parkinson’s disease and Alzheimer’s disease. Our hypothesis-generating results from gene expression profiling of BECs may have relevance to these other neurological diseases. The selective COX-2 inhibitor celecoxib is clinically available and may be studied further as a potential treatment for SAH.

## Materials and methods

All protocols were approved by the Animal Care Committee at St. Michael’s Hospital, Toronto, ON, Canada. All experiments were performed in accordance with Animal Care Committee guidelines and regulations. Reporting of the results follow ARRIVE guidelines. Full details of materials and methods are described in the supplement.

### Animals

Tg(*Tie2*GFP)287Sato/J transgenic mice (Jackson Laboratory, Bar Harbor, ME) were used in the BBB assessment with cadaverine dye experiments and isolation of Tie2 + Pdgfrb- BECs. All other experiments used wild-type FVB/NJ mice (Jackson Laboratory, Bar Harbor, ME).

### Prechiasmatic injection SAH model

Details of the SAH model have been described^48^. Mice were placed under anaesthesia using inhaled isoflurane and then turned prone with the skull fixed in a stereotactic apparatus. A mid-line dorsal incision over the skull was created and a burr hole made 1 mm anterior to the olfactory sulcus and 1 mm to the left of midline. A 27-gauge needle was inserted through the burr hole at 37.5 degrees from the vertical plane down to the skull base, after which 80 microL of whole blood obtained from a littermate was injected. The sham procedure involved needle insertion without blood injection. After 2 min, the needle was removed and the incision closed. The mice were allowed to recover for 24 h to 48 h after the procedure.

### Neurobehavioural assessments

Mice were assessed on the Modified Garcia Score at 24 h and 48 h by 2 assessors in a blinded fashion^49^.

### Histological assessment of brain injury

Coronal slices of mouse brains 24 h after SAH or sham procedure were fixed and stained for fluoro-jade B (Histo-Chem Inc., Jefferson, AK) and caspase 3 (BD Pharmingen, San Diego, CA) to detect degenerated and apoptotic neurons respectively.

### Blood brain barrier assessment

Fluorescent cadaverine dye was administered via intraperitoneal injection 2 h prior to transcardiac perfusion. Microscopy was used to image whole brains and coronal brain slices.

### Brain endothelial cell isolation

Cell suspensions were created from left cerebral hemispheres via mechanical and enzymatic dissociation. Endothelial cells were then isolated by two protocols: (1) Tie2 + Pdgfrb- endothelial cells isolated by fluorescence-activated cell sorting (FACS), and (2) CD45–CD31 + endothelial cells by magnetic bead sorting (Supplementary Fig. S10). Purity and viability of cell suspensions were assessed by flow cytometry.

### RNA extraction and microarray analysis

RNA was extracted from endothelial cell suspensions using commercially available kits including RNeasy micro kit (Qiagen Inc., Venlo, Netherlands) and Arcturus PicoPure RNA isolation kit (Applied Biosystems, Foster City, CA). Isolated RNA was then spiked with 0.25 ng of exogenous luciferase plasmid (pSP-luc + NF cloning vector, U47123.2) and then underwent reverse transcription polymerase chain reaction. Isolated RNA from CD45–CD31 + BECs was amplified with Ovation Pico WTA System V2 (NuGEN, Redwood City, CA). Amplified RNA was then labeled with Encore Biotin Module (NuGEN, Redwood City, CA) and hybridized to Affymetrix Mouse Gene 2.0 ST Arrays (Affymetrix, Santa Clara, CA). Analysis was completed with R statistical software (R Foundation for Statistical Computing, R version 3.2.2). Gene set enrichment analysis (GSEA) with Gene Set Knowledgebase (“gskb”) was used to identify enriched pathways^50^. The dataset has also been deposited in NCBI’s Gene Expression Omnibus (GEO) and is accessible by accession number GSE155137 (https://www.ncbi.nlm.nih.gov/geo/query/acc.cgi?acc=GSE155137).

### Celecoxib treatment

Celecoxib (10 mg/kg, Sigma-Aldrich Inc., St. Louis, MO) dissolved in 100 µL of 50:50 DMSO (dimethyl sulfoxide):normal saline was administered via intraperitoneal injection 30 min and 12 h after SAH or sham procedure. Vehicle consisted of 100 µL of 50:50 DMSO:normal saline solution.

### Statistics

Unless otherwise stated, data were presented as means ± standard errors. Comparisons between 2 groups were made using Student’s t test for parametric data and Mann–Whitney U test for non-parametric data. Comparisons between multiple groups were made using ANOVA with Holm-Sidak post hoc correction for parametric data and Kruskal–Wallis test for non-parametric data. Aside from the microarray analysis (R Foundation for Statistical Computing, R version 3.2.2), all statistical analyses were performed using Prism 8 (Graphpad Software, San Diego, CA).

### Conference presentation

Portions of this work have been presented at the following conferences: American Association of Neurological Surgeons (AANS) annual meeting in Chicago, IL (May 1, 2016); Canadian Neurological Sciences Federation (CNSF) annual congress in Quebec City, QC (June 22, 2016); and the International Stroke Conference (ISC) in Houston, TX (Feb 23, 2017). This results of this study have previously been published in the form of a PhD thesis at the University of Toronto.

## Supplementary Information

Supplementary Information 1.

Supplementary Table S1.

Supplementary Table S2.

Supplementary Table S3.

## Data Availability

The dataset generated from this study can be found in Supplementary Table S3. The dataset has also been deposited in NCBI’s Gene Expression Omnibus (GEO) and is accessible by accession number GSE155137 (https://www.ncbi.nlm.nih.gov/geo/query/acc.cgi?acc=GSE155137).
