# Supplementary material for: Gene expression profiling of brain endothelial cells after experimental subarachnoid haemorrhage
Source: Sci Rep. 2021 Apr 9;11:7818. doi: 10.1038/s41598-021-87301-z (PMC8035152; doi:10.1038/s41598-021-87301-z)
Supplement: Supplementary file 1 — Supplementary Information 1. [file 41598_2021_87301_MOESM1_ESM.docx]

Scientific Reports

Supplementary Information

Gene expression profiling of brain endothelial cells after experimental subarachnoid haemorrhage

Michael K. Tso,^1,2^ Paul Turgeon,^3^ Bert Bosche,^2,4, 5, 6^ Charles K Lee,^2^ Tian Nie,^2^ Josephine D’Abbondanza,^2^ Jinglu Ai,^2^ Philip A Marsden,^3^ R Loch Macdonald^2^

^1^Division of Neurosurgery, University of Calgary, Calgary, AB

^2^Division of Neurosurgery, St. Michael’s Hospital, Keenan Research Centre for Biomedical Science and the Li Ka Shing Knowledge Institute of St. Michael’s Hospital, University of Toronto, Toronto, ON

^3^Division of Nephrology, University of Toronto, Toronto, ON

^4^Department of Neurocritical Care, Neurological and Neurosurgical First Stage Rehabilitation and Weaning, MediClin Clinic Reichshof, Reichshof-Eckenhagen, Germany ^5^Institute of Neurophysiology, University of Cologne, Cologne, Germany

^6^Department of Neurology, University of Duisburg-Essen, Essen, Germany

**Supplementary Methods**

**SAH model**. Mouse strains utilized included 12-week-old male FVB/NJ mice (Jackson Laboratory) and 12-15-month-old male and female Tg(*Tie2*GFP)287Sato/J mice (Jackson Laboratory). These latter mice have an FVB background but also express green fluorescent protein (GFP) driven by the endothelial cell specific *Tie2* promoter, allowing blood vessels to be visualized *in vivo*. A total of 110 FVB/NJ mice were used and 36 Tg(*Tie2*GFP)287Sato/J mice were used. Mice were housed up to 5 mice per cage with standard chow and water *ad libitum* and 12 h /12 h light/dark cycles. Mice were randomized to SAH or sham procedure. Anesthesia was induced with inhaled 3% isoflurane (Fresnius Kabi) with 1 L oxygen flow. Hair over the dorsal skull is shaved and prepped with 10% povidone-iodine topical solution (Laboratoire Atlas Inc.). Mice also receive subcutaneous injection of buprenorphine (0.2 mg/kg) and lubricating ophthalmic ointment (Refresh Lacri-Lube, Allergan) over the eyes. Mice were placed prone and skull fixated using a stereotactic frame. Using the prechiasmatic blood injection mouse model, which reliably creates a blood clot along the ventral brain surface and basal cisterns like what is seen in ruptured anterior circulation aneurysms.^1^ After a midline skin incision over the dorsal skull, a burr hole was created with a high-speed drill (WE243 rechargeable mini engraver 2, Wecheer Industrial Co., Ltd.) with a 1 mm round stainless steel bur (1RF 007, 330 104 001 001, Hager & Meisinger GmbH) 1 mm to the left of midline and 1 mm rostral to the fissure separating the olfactory bulbs and cerebral cortex, in order to avoid major sinus bleeding. Using a 27G Whitacre pencil point spinal needle (BD Medical) oriented at 37.5^o^ from the vertical plane, 80 µL of littermate arterial blood was injected over 45 sec. This transarterial blood was freshly obtained via transcardial puncture under intraperitoneal ketamine/xylazine anesthetic. After injection, the spinal needle was left in place for 2 min to allow the blood to clot and to minimize reflux through the burr hole. Upon removal of the spinal needle from the skull, bone wax (Ethicon) was used to plug the burr hole. Sham procedure involved burr hole creation and needle insertion without blood injection. Intraoperative cerebral blood flow (CBF) was measured by laser doppler flow meter (BLF 21, Transonic Systems Inc.) placed over the contralateral right parietal skull with measurements made 10 min prior to and post-SAH induction at 2-minute intervals. Mice were placed on a homeothermic blanket (Harvard Apparatus) with rectal probe in place and set to maintain 37 ^o^C. Postoperative subcutaneous buprenorphine (0.2 mg/kg) was administered twice daily. Weights were measured preoperatively and at 24 h and at 48 h, if applicable, after SAH/sham procedure.

**Neurobehavioural Assessment**. Global neurobehavioural assessments were performed at 24 h and 48 h after SAH/sham procedure using the Modified Garcia Score (MGS, maximum score 18 indicative of normal behavior) performed by 2 blinded observers.^2^ As a measure of activity level, the time to touch 3 separate walls with 2 paws simultaneously was recorded for up to 5 min (the first component of the MGS). If the mouse failed to touch 3 separate walls, then a time of 5 min was recorded. For comparison between 2 groups, the non-parametric Mann-Whitney U test was used. For comparison between more than 2 groups, the non-parametric Kruskal-Wallis test was used.

**Celecoxib Treatments**. Mice received intraperitoneal injections of celecoxib (PZ0008, Sigma-Aldrich Inc.) at a dose of 10 mg/kg, dissolved in a 100 μL volume of 1:1 dimethyl sulfoxide (DMSO) : normal saline (NS). Dosing is equivalent to adult human dose of 100 mg twice daily, based on body surface area dosing conversion.^3^ Doses of celecoxib or vehicle were administered 30 min and 12 h after SAH/sham induction. Vehicle injection consisted of 100 μL of 1:1 DMSO : NS. These injections were not blinded.

**BBB permeability assay**. BBB permeability studies were performed in 12-15 month old, male and female Tg(*Tie2*GFP)287Sato/J mice (SAH 24 h/48 h vs. Sham 24 h/48 h, *n* = 4 per group) and 12 week old male FVB/NJ mice (SAH +/- vehicle/celecoxib vs. sham +/- vehicle/celecoxib, *n* = 3 per group). Mice were administered intraperitoneal injections of cadaverine conjugated to Alexa Fluor 555 (250 μg / 25 g mouse, dissolved in 100 μL sterile phosphate-buffered saline (PBS), A30677, Life Technologies Inc) 2 h prior to transcardial perfusion. Cadaverine is the decarboxylation product of the amino acid lysine, typically present in decaying tissue but also present in small amounts in live animals.^4^ The conjugated dye is a water-soluble tracer with molecular weight of approximately 950 Da. Transcardial perfusion was performed after induction of general anesthesia with intraperitoneal ketamine hydrochloride (200mg/kg) and xylazine hydrochloride (20 mg/kg). The descending aorta was clamped prior to left ventricular puncture with a 23G needle. Subsequently, the right atrium was punctured. Perfusion was performed with a pressurized tubular system with the sphygmomanometer set to maintain 100mmHg. Brains were extracted and placed in ice-cold PBS. The right kidney was also extracted, to assess the degree of systemic absorbance of the cadaverine dye. Successful absorbance of the cadaverine dye was also confirmed by the urine demonstrating a bright pink colour. Whole brains and kidneys were imaged with a Leica DFC365FX camera (Leica Microsystems Inc.) mounted to a Leica M205FA upright fluorescent microscope (Leica Microsystems Inc.) with GFP and mCherry filter cubes. Brains were imaged in the ventral, dorsal, left lateral, and right lateral positions under the same exposure time, gain and intensity settings. Brains were then fixed in 4% paraformaldehyde (PFA) at 4 ^o^C for 6 h followed by coronal sectioning at 50 µm thickness using the Leica VT1200S vibratome (Leica Microsystems Inc.). These coronal slices were placed on microscope slides and the nuclei were stained with DAPI (4’,6-diamidino-2-phenylindole, Sigma-Aldrich) for 15 min followed by several washes. Aqueous-based CC/mount (Sigma-Aldrich) was added to the specimen slides and then cover-slipped. Confocal images of left and right sides of coronal sections were taken using the Zeiss LSM700 confocal microscope (Carl Zeiss Canada LTD.) in the ventral medial, ventral lateral, lateral, and dorsal regions under the same exposure time, gain and power settings. For quantification, whole brain ventral images were analyzed with Image J (NIH). Coronal slices were quantified using the triangle algorithm for auto-thresholding and “analyze particle” function from Image J for identifying positive cells.

**Brain endothelial cell isolation protocol #1: AutoMACS Method (Supplementary Fig. S10)**. Twelve week old male FVB mice (*n* = 4 per group for microarray studies, *n* = 3 per group for celecoxib studies) were anesthetized with intraperitoneal ketamine hydrochloride (200 mg/kg) and xylazine hydrochloride (20 mg/kg) and subsequently transcardially perfused with ice-cold sterile PBS (14190-144, gibco by Life Technologies) for 2 min, similar to the perfusions for the cadaverine-based studies described above. Brains were extracted and placed in a petri dish with ice-cold sterile PBS. The cerebellum and brainstem/diencephalon were removed by sharp dissection. The cerebral hemispheres were sharply separated. Because the majority of cadaverine leakage occurred in the left ventral region, only the left cerebral hemisphere was utilized for the brain endothelial cell (BEC) isolation protocol to maximize the signal magnitude. The meninges of the left cerebral hemisphere were dissected off and the brain tissue was gently rolled on sterile filter paper to remove pial vessels from the dorsal brain surface, and Circle of Willis arteries and subarachnoid blood clot from the basal surface. The purpose of these maneuvers is to minimize inclusion of endothelial cells from the arteries and larger arterioles and to minimize *ex vivo* activation of isolated endothelial cells by the subarachnoid blood clot. The left cerebral hemisphere was reproducibly dissociated by a combination of mechanical means with the brain dissociation protocol from the gentleMACS system using C-tubes (130-093-237, Miltenyi Biotec) and by enzymatic means with the papain-based Neural Tissue Dissociation Kit (130-092-628, Miltenyi Biotec). Because our cell surface marker of interest, Pecam1/CD31, is somewhat sensitive to papain enzymatic digestion, we utilized 10% of the usual volume of stock papain enzyme (5 μL instead of 50 μL). The left cerebral hemisphere was dissociated in 2 mL volume of buffer containing 0.067 mM of β-mercaptoethanol (M6250, Sigma-Aldrich) and enzyme mix as per the instruction manual. After a series of incubation and mechanical dissociation steps, the suspension was then passed through a 70 μm nylon cell strainer (352350, Falcon), washed with sterile Hank’s Balanced Salt Solution without calcium or magnesium (HBSS-/-, 14175-095, gibco by Life Technologies), and centrifuged at 300 g for 10 min. The supernatant was discarded, and pellet was resuspended with 1,800 μL of buffer containing HBSS-/- and 0.5% bovine serum albumin (BSA, 30% stock solution, A9576, Sigma-Aldrich). All subsequent steps utilized the HBSS-/- with 0.5% BSA as buffer. The brain suspension was incubated with 200 μL of myelin removal beads II for 15 min (IgM mouse anti-mouse, 130-096-733, Miltenyi Biotec). After another wash and centrifugation at 300 g for 10 min, the pellet was resuspended with 2 mL of buffer and transferred to 5 mL polypropylene round-bottom tubes (352063, Falcon). Using the “deplete” protocol from the autoMACS Pro Separator (130-092-545, Miltenyi Biotec) and fresh pack of autoMACS separation columns (130-021-101, Miltenyi Biotec), the brain suspension was depleted of myelin, creating a 4mL output of “Total Cell Suspension.” For all autoMACS magnetic sorting, we utilized the autoMACS Running Buffer which contains BSA (130-091-221, Miltenyi Biotec). This suspension was centrifuged at 300 g for 10 min. After supernatant removal, the cell pellet was resuspended with 85 μL of buffer and 5 μL of Fc receptor blocker (CD16/32, IgG rat anti-mouse, 553141, BD Pharmingen) for 5 min, followed by incubation with 10 μL of CD45 (leukocyte marker) microbeads (IgG2b rat anti-mouse, 130-052-301, Miltenyi Biotec) for 15 min. After a wash and centrifugation at 300 g for 5 min, the cell pellet was resuspended with 500 μL of buffer. Using the “depleteS” protocol from the autoMACS Pro Separator, the total cell suspension was depleted of CD45, creating 2.5 mL output of CD45- cell suspension. A CD45 depletion step was essential as leukocytes also express CD31 which could confound results. This suspension was centrifuged at 300 g for 10 min and supernatant was removed. The cell pellet was resuspended with 80 μL of buffer and incubation with 20 μL of CD31/Pecam1 (Platelet-endothelial cell adhesion molecule 1, endothelial marker) microbeads (IgG2a rat anti-mouse, 130-097-418, Miltenyi Biotec) for 15 min. After a wash and centrifugation at 300 g for 5 min, the cell pellet was resuspended with 500 μL of buffer. Using the “posseld” protocol from the autoMACS Pro Separator which utilizes 2 magnetic columns and creates a small elution volume ideal for antigen expression of less than 5% of cells, the CD45- cell suspension was enriched for CD31, creating 500 μL output of CD45-CD31+ cells or “Endothelial Cell Suspension.” The negative fraction CD45-CD31- cell suspension was also collected or “Parenchymal Cell Suspension.” Quality control included assessment of cell viability by uptake of intracellular dye propidium iodide (PI, 1:1,000, P4864, Sigma-Aldrich) on flow cytometry (MACSQuant, Miltenyi Biotec). Endothelial cell suspension (CD45-CD31+) purity was assessed by flow cytometry using conjugated antibodies (Rat anti-mouse CD31-FITC, 553372, BD Pharmingen; Rat anti-mouse CD45-APC, 17-0451-82, eBioscience) and their respective rat isotype controls (IgG2a-FITC, 553929, BD Pharmingen; IgG2a-APC, 553932, BD Pharmingen). Typical viability was > 80% and CD45-CD31+ purity > 90%. All buffers (HBSS-/-, HBSS-/- with 0.5% BSA) were kept on ice, equilibrated with oxygen probes (95% O_2_, 5% CO_2_) and pH adjusted to 7.4. All steps after enzymatic digestion were performed at 4 ^o^C. Two independent biological samples were processed simultaneously per day (typically one SAH left hemisphere and one Sham left hemisphere), and the order of processing alternated by day (e.g. SAH followed by Sham one day, Sham followed by SAH the next day, etc.). Time from transcardial perfusion to placement in RLT extraction buffer was typically 3.5 h.

*RNA Extraction*. Endothelial cell suspension derived from the autoMACS isolation protocol (CD45-CD31+ cells), as well as the total cell and parenchymal cell suspensions, was centrifuged for 5 min at 300 g at 4 ^o^C. The supernatant was carefully aspirated followed by addition of 75 μL of RLT extraction buffer (Qiagen Inc) containing 1% β-mercaptoethanol. Addition of 0.025 ng of luciferase mRNA plasmid (pSP-luc+NF cloning vector, U47123.2) in a 10 μL volume was performed for determining first strand synthesis and amplification efficiencies of each sample. Samples were then transferred to a QIAshredder spin column (79654, Qiagen Inc) and centrifuged at 25,000 g for 2 min to ensure complete cell lysis. Total RNA was extracted using the RNeasy micro kit (74004, Qiagen Inc) as per the provided instructions manual. On-column DNase I was utilized (10 μL reconstituted stock DNase I, 70 μL RDD buffer, 79254, Qiagen Inc). For RNA elution, 15 μL volume of RNase-free water was utilized. RNA quantity and quality were assessed using Nanodrop 2000 (Thermo Scientific) and Agilent 2100 Bioanalyzer with the RNA 6000 Pico Reagents Part I Series II (Agilent Technologies). Remaining RNA samples were stored at -80 ^o^C until further downstream gene expression assays. Typical RNA concentrations were 0.5-2 ng/μL with RNA integrity number (RIN) > 7.0.

**Brain endothelial cell isolation protocol #2: FACS Method (Supplementary Fig. S10)**. An alternative strategy of fluorescent-activated cell sorting (FACS protocol) was utilized in 12-15-month-old male Tg(Tie2GFP)287Sato/J mice (*n* = 4 per group) to isolate BECs with significant modifications to a previously reported isolation strategy [Daneman 2010]. Brain extraction and dissociation steps utilized the papain-based neural tissue dissociation kit and gentleMACS systems, like the autoMACS method described above. However, in this protocol, the full 50 µL volume of papain was used. Magnetic-based myelin depletion using the autoMACS Pro Separator was utilized like above, creating 4 mL output of “Total Cell Suspension.” We incubated this cell suspension with *Pdgfrb* (Platelet-derived growth factor receptor beta, a pericyte marker) antibodies conjugated to the fluorescent dye allophycocyanin (APC) (1:1,000, A18383, molecular probes by Life Technologies) for 10 min at 4 ^o^C. PI (1:1,000) was added and cells were sorted by FACS with BD FACSAria III Cell Sorter (BD Biosciences) at 4 ^o^C. Non-fluorescent FVB mouse brain homogenates were used to gate for GFP^+^ cells. Total cell suspension derived from Tg(Tie2GFP)287Sato/J mice but not stained with Pdgfrb-APC was used to gate for APC^+^ cells. Gating parameters were selected for live single cells that were GFP^+^ and APC^-^ (Tie2^+^Pdgfrb^-^), which were sorted into 200 µL of HBSS-/- with 0.5% BSA and 10% 0.5 M ethylenediaminetetraacetic acid (EDTA). For BEC purity assessment, a small aliquot of cell suspension underwent a second confirmatory sort. FACS time was typically 1 h. Time from transcardial perfusion to placement in RNA extraction buffer was typically 3 h. Only a single biological replicate was processed per day. FACS analysis performed with BD FACSDiva Software Version 8.0.1 (BD Biosciences).

*RNA Extraction*. Total RNA from BECs derived from the FACS method (Tie2+Pdgfrb-) was extracted using the Arcturus PicoPure RNA isolation kit (KIT0204, Applied Biosystems). After obtaining FACS-derived Tie2+Pdgfrb- cells, the cell suspension was centrifuged at 1,000 g for 10 min at 4 ^o^C. The supernatant was carefully removed followed by addition of 100 µL of RNA extraction buffer containing 1% β-mercaptoethanol. This mixture was incubated at 42 ^o^C for 30 min with gentle agitation. RNA extraction was performed according to the instruction manual of the Arcturus PicoPure RNA isolation kit. On-column DNase was added (10 µL of DNase stock, 30 μL of RDD buffer per sample). For RNA elution, 11.5 μL of elution buffer was utilized. RNA quantity and quality were assessed using Nanodrop 2000 and Agilent 2100 Bioanalyzer like above. Remaining RNA samples were stored at -80^o^C until further downstream gene expression assays. Typical RNA concentrations were 0.5-1 ng/μL. However, the RINs from FACS-derived BECs were generally poor (range 2.0-7.0) compared to RNA from autoMACS-derived BECs. Hence, only the RNA from autoMACS-derived BECs (CD45-CD31+ cells) were utilized for amplification and microarray analysis.

**Microarray**. Equal quantities of extracted RNA derived from CD45-CD31+ endothelial cells (*n* = 4 per group, with each sample being a single unique biological replicate without pooling) were amplified using the Ovation Pico WTA System V2 (NuGEN), which utilizes unique RNA-DNA hybrid oligo-dT and random hexamer primers for linear amplification. We used 4 biological replicates per group as that was a reasonable minimum sample size to obtain potential differences in gene expression. Subsequently, 5.0 μg of cDNA was fragmented and labeled using the Encore Biotin Module (NuGEN) and hybridized to Affymetrix Mouse Gene 2.0 ST Arrays for 18 h at 45 ^o^C at 60 revolutions per minute (RPM). This microarray platform utilizes about 21 perfect-match oligo-probes for each of 41,345 probe-sets representing 35,240 RefSeq transcripts including 26,191 well-established protein-coding genes and 3,391 well-established non-coding genes, which includes approximately 2000 long non-coding RNAs (lncRNAs). Arrays were then washed using GeneChip Fluidics Station P450 and scanned with Affymetrix GeneChip Scanner 7G. Hybridization controls were similar across all arrays. Microarray hybridizations were performed with the assistance of the Princess Margaret Genomics Centre (PMGC, Toronto, Canada, www.pmgenomics.ca). Microarray raw intensity values were normalized using the robust multi-array average (RMA) algorithm with R statistical software (R Foundation for Statistical Computing, R version 3.2.2), with the “oligo” package derived from Bioconductor version 3.2.^5^ Clustering of sample groups were determined using unsupervised hierarchical clustering, Pearson’s correlation and principal-component analysis (PCA). Differentially expressed genes (SAH vs. Sham) were identified using independent 2-tailed t-tests with multiple testing correction via the Benjamini-Hochberg false discovery rate (FDR) method (significance level of 0.05).^6^ Heatmaps were generated using the “gplots” package. Volcano plot was generated using the “plot” function and “calibrate” package. PCA plot was generated using the GeneSpring v13.1 software. Gene Set Enrichment Analysis (GSEA) was used to identify pathways relevant to the patterns of gene expression observed.^7^ Mouse-specific gene pathways were interrogated using the “gskb” (Gene Set Knowledgebase) package, which contains 33,261 gene sets from 40 sources involving gene ontology (GO), chromosome location, metabolic pathways, curated pathways, and target genes of transcription factors. See Supplementary Table S1 for list of significantly upregulated and downregulated after experimental SAH. See Supplementary Table S2 for the full gene-probe set data from microarray.

The dataset has also been deposited in NCBI’s Gene Expression Omnibus (GEO) and is accessible by accession number GSE155137 (<https://www.ncbi.nlm.nih.gov/geo/query/acc.cgi?acc=GSE155137>).

**RT-PCR Validations**. Microarray results were validated by real-time polymerase chain reaction (RT-PCR) using amplified cDNA samples derived from CD45-CD31+ cells. Unamplified total RNA derived from CD45-CD31+ and Tie2+Pdgfrb- was converted to cDNA using Superscript III First-Strand Synthesis SuperMix for qRT-PCR (11762050, Invitrogen by Life Technologies). Primers were designed using Oligo Primer Analysis Software version 7 (Molecular Biology Insights, Inc.). Primers were obtained from IDT (Integrated DNA Technologies) for the following genes: luciferase (pSP-luc+NF cloning vector), *Actb* (β-actin), *Ppia* (Cyclophilin A), *Ptgs1* (Cox1), *Ptgs2* (Cox2), *Angpt1*, *Angpt2*, *Tie2*, *Vegfr2*, *Mfsd2a*, *Pecam1* (CD31), *Cdh5* (VE-Cad), *Vcam1*, and *Klf2*. Equal amounts of cDNA underwent RT-PCR with technical triplicates with SYBR Green PCR master mix (4309155, Applied Biosystems) and ABI Prism 7900 Real-Time PCR System (Applied Biosystems), undergoing 40 cycles of 15 s at 95 ^o^C followed by 1 min at 60 ^o^C. Relative mRNA expression was determined using ΔΔCt method with the sham sample as the experimental control and β-actin as house-keeping gene [Schmittgen 2008]. First-strand synthesis efficiency was determined by dividing the measured absolute copy numbers of luciferase plasmid using a standard curve from RT-PCR with the known quantity of luciferase plasmid added at time of RNA extraction. Similarly, amplification efficiency was determined by dividing the measured absolute copy numbers of luciferase plasmid from amplified CD45-CD31+ cDNA with the absolute copy numbers of luciferase plasmid from unamplified CD45-CD31+ cDNA. See Supplementary Table S3 for list of RT-PCR primers used.

**Immunofluorescence**. Mice were transcardially perfused with PBS for 2 min followed by 4% PFA for 5 min at a constant pressure of 100mmHg. Brains were extracts and fixed in 4% PFA at 4 ^o^C for 24 h. A coronal cut was made 4mm from the anterior pole. Brain tissue were then sequentially dehydrated using the Leica TP 1020 Automatic Tissue Processor (Leica) followed by paraffin embedding. Coronal slices 5µm thick were created using a microtome. For immunofluorescence histology, slides with coronal brain slices were gradually rehydrated, followed by heat-mediated antigen retrieval. Specimens were then blocked with 10% goat serum. Primary antibodies were incubated for 1 hour at room temperature or overnight at 4 ^o^C. After washes, secondary antibodies were incubated for 1 hour at room temperature. After more washes, specimens were incubated with DAPI for 15 min, followed by more washes, and mounting with aqueous media and glass coverslip. Images were taken using the Olympus inverted fluorescent microscope and Zeiss confocal microscope. Primary antibodies: rabbit anti-mouse Cox2 (Abcam, 1:200), mouse anti-mouse NeuN (Millipore, 1:400, neuronal marker), rabbit anti-mouse caspase3 (BD Pharmingen, 1:300). Secondary antibodies: goat anti-rabbit Alexa Fluor 568 (Life Technologies), goat anti-mouse Alexa Fluor 488 (Life Technologies). Image processing included using the triangle algorithm for auto thresholding and the “analyze particles” function on Image J.

**Fluoro-jade B staining.** Paraffin-embedded coronal slices were gradually deparaffinized and rehydrated. Microscope slides were placed in 0.06% KMnO_4_ (Sigma-Aldrich) for 8 min and then rinsed multiple times with deionized water. Microscope slides were then placed in 0.001% fluoro-jade B solution (Histo-Chem Inc., Jefferson, Arkansas) for 30 min. Slides were then washed, dried, and placed in xylene prior to being cover-slipped with non-aqueous DPX mounting medium (Sigma-Aldrich). Images were taken using the Olympus inverted fluorescent microscope. Image processing included using the triangle algorithm for auto thresholding and the “analyze particles” function on Image J.

**Enzyme-linked immunosorbent assay (ELISA)**. Whole blood samples were obtained from mice via cardiac puncture at the time of transcardial perfusion. Serum samples were obtained by allowing whole blood samples to clot at room temperature for 2 hours, followed by centrifugation at 2,000 g x 20 min, and storage of the supernatant at -20 ^o^C until the time of usage. Mouse brain tissue was extracted at the time of transcardial perfusion, snap frozen in liquid nitrogen and stored at -80degC until the time of homogenization. Brain tissue was homogenized in 1 mL of sterile phosphate-buffered saline and centrifuged at 5,000 g x 5 min, with the supernatant subsequently transferred to a separate tube and stored at -20 ^o^C until the time of usage. Brain tissue homogenates and serum samples were assessed using commercially-available ELISA kits for Tie2 (Mouse Tie-2 Quantikine ELISA kit, MTE200, R&D Systems), Angpt1 (Mouse Angiopoietin-1 ELISA kit, LS-F13065, LSBio Inc), and Angpt2 (Mouse/rat Angiopoietin-2 Quantikine ELISA kit, MANG20, R&D Systems). Serum samples were diluted 2-fold, 40-fold, and 40-fold for the Tie2, Angpt1, and Angpt2 ELISA experiments, respectively. Brain homogenates were diluted 2-fold, 1-fold, and 10-fold for the Tie2, Angpt1, and Angpt2 ELISA experiments, respectively. Absorbance values were detected by spectrophotometer (SpectraMax M5e, Molecular Devices, Sunnyvale, CA) and analyzed with SoftMax Pro 5 (Molecular Devices, Sunnyvale, CA).

**Supplementary Figures**

**
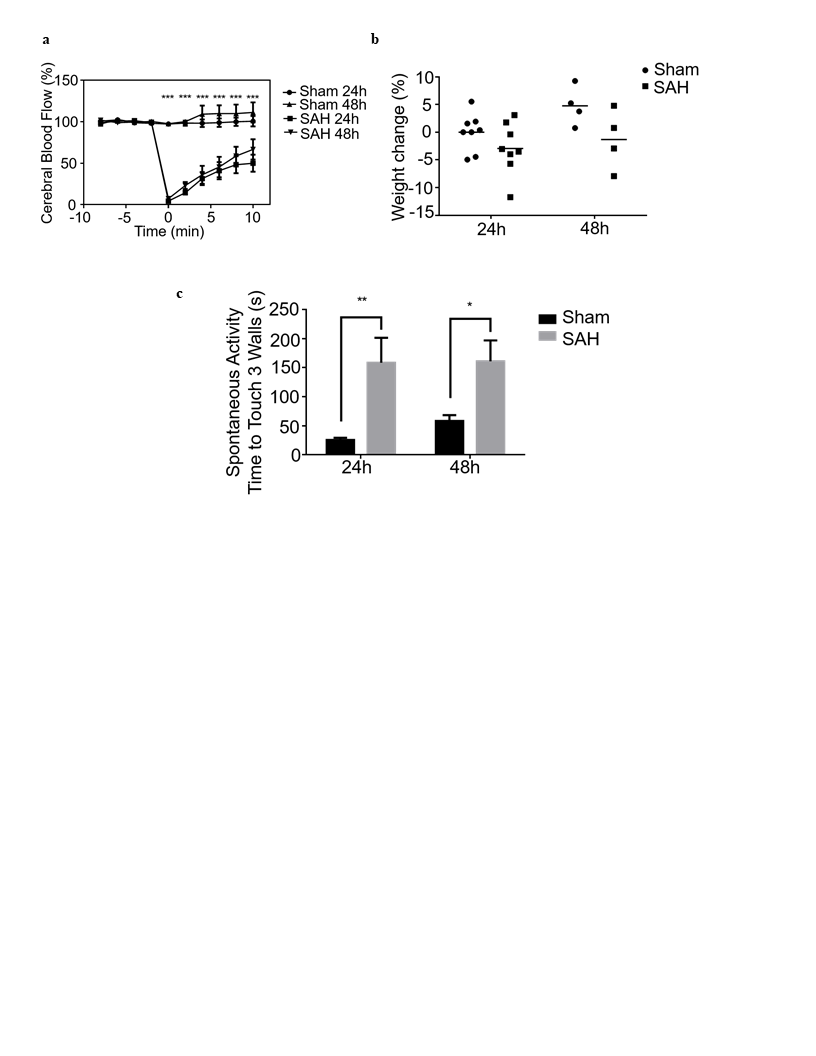
**

**Supplementary Figure S1.** **Experimental SAH model.** (**a**) Perioperative cerebral blood flow (CBF) measurements over time with SAH induction (or insertion of needle without injection of blood in sham procedure) occurring at time 0. (**b**) Percent weight change 24 h and 48 h after SAH or sham procedure (**c**) Spontaneous activity assessment 24 h and 48h h after SAH or sham procedure. *n* = 4 per group. Data presented as means ± SEM (Standard error of the mean). Two-way ANOVA with Holm-Sidak *post hoc* correction (CBF). Kruskal-Wallis test (Weight change, spontaneous activity). **p* < 0.05, ***p* < 0.01, ****p* < 0.001 (relative to Sham 24 h or Sham 48 h).


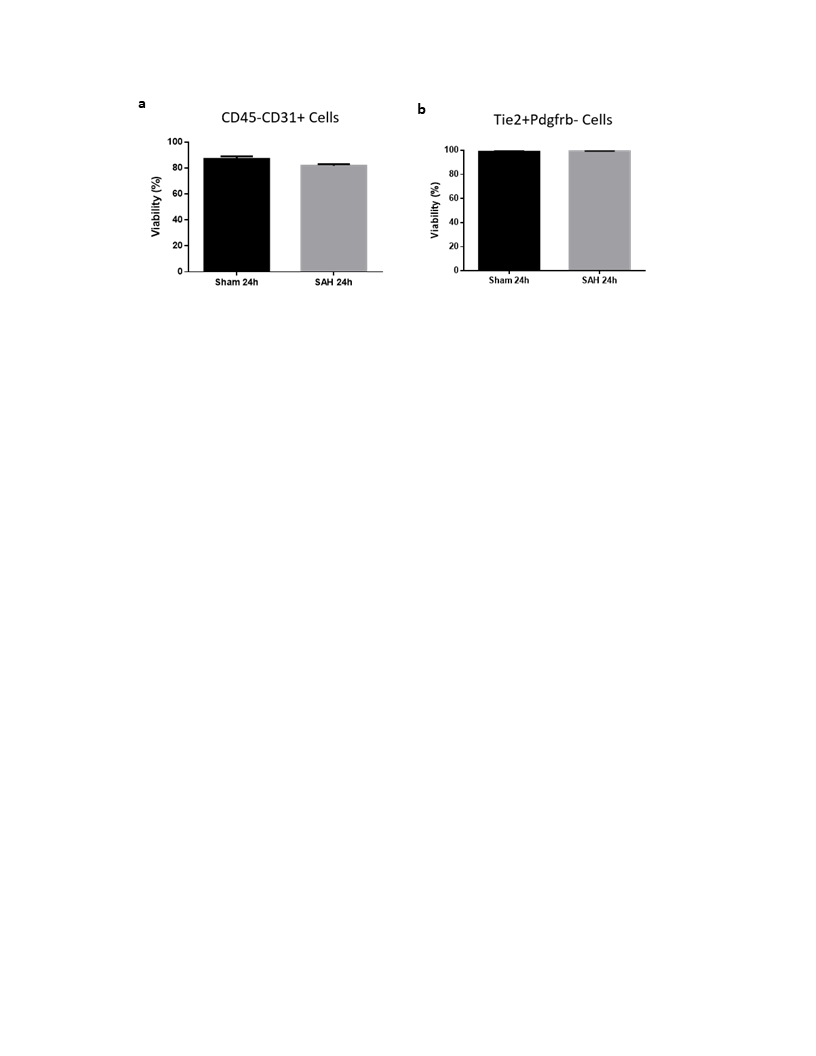


**Supplementary Figure S2. Viability of isolated brain endothelial cells**. (**a**) Viability of CD45-CD31+ endothelial cells 24 h after SAH or sham procedure. (**b**) Viability of Tie2+Pdgfrb- endothelial cells 24 h after SAH or sham procedure. Data presented as means ± SEM.


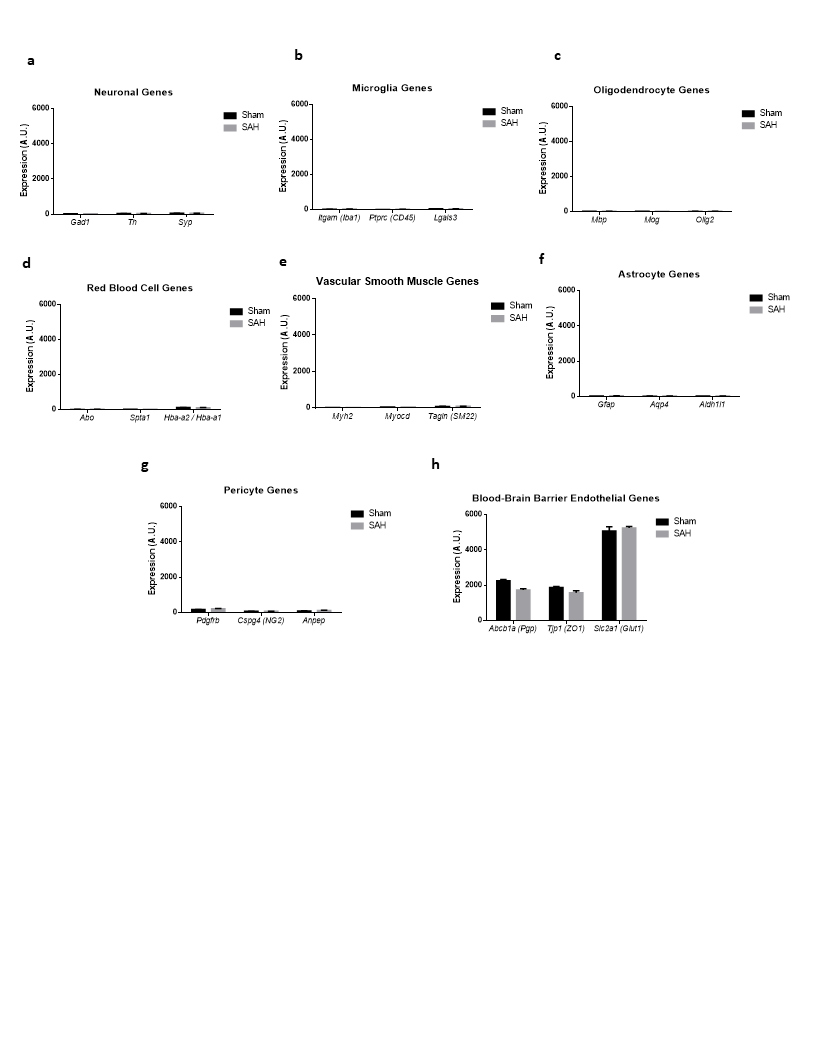


**Supplementary Figure S3. Expression levels of genes characteristic of different cell types**. **(a-h)** Expression levels based on microarray intensity values characteristic of different cell types including neurons, microglia, oligodendrocytes, red blood cells, vascular smooth muscle cells, astrocytes, pericytes, and blood brain barrier endothelial cells. *n* = 4 per group. Data presented as means ± SEM. Abbreviations: *Abo*: Transferase A, alpha 1-3-N-acetylgalactosaminyltransferase, transferase B, alpha 1-3-galactosyltransferase; *Aldh1l1*: Aldehyde dehydrogenase 1 family, member L1; *Anpep*: Alanyl (membrane) aminopeptidase; *Aqp4*: Aquaporin 4; *Cspg4*/NG-2: Chondroitin sulfate proteoglycan 4; *Gad1*: Glutamate decarboxylase 1; *Gfap*: Glial fibrillary acidic protein; *Hba-a2/Hba-a1*: Hemoglobin alpha, adult chain 1 / hemoglobin alpha, adult chain 2; *Itgam*/Iba1: Integrin alpha M; *Lgals3*/Galectin3: Lectin, galactose binding, soluble 3; *Mbp*: Myelin basic protein; *Mog*: Myelin oligodendrocyte glycoprotein; *Myh2*: Myosin, heavy polypeptide 2; *Myocd*: Myocardin; *Olig2*: Oligodendrocyte transcription factor 2; *Pdgfrb*: Platelet derived growth factor receptor, beta polypeptide; *Pecam1*/CD31: Platelet/endothelial cell adhesion molecule 1; *Ptprc*/CD45: Protein tyrosine phosphatase, receptor type, C; *Spta1*: Spectrin alpha, erythrocytic 1; *Syp*: Synaptophysin; *Tagln3*/SM22: Transgelin 3; *Tek*/*Tie2*: Tunica intima endothelial kinase 2; *Th*: Tyrosine hydroxylase; *Tjp1*/ZO-1: Tight junction protein 1 / Zonula occludens 1.


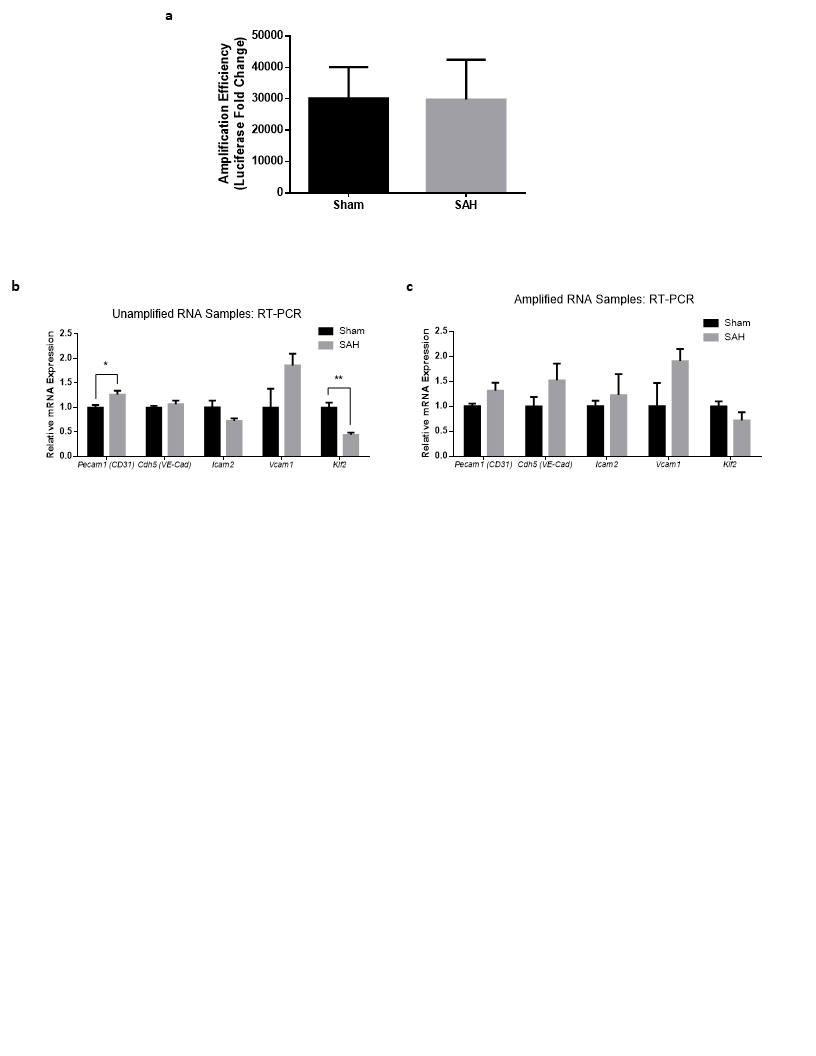


**Supplementary Figure S4. Amplification of RNA**. (**a**) Amplification efficiencies 24h after SAH or sham procedure based upon fold change in luciferase transcript level measurement. (**b-c**) Expression level of CD45-CD31+ endothelial genes before and after RNA amplification in CD45-CD31+ endothelial cells. *n* = 4 per group. Data presented as means ± SEM. *t*-test, **p* < 0.05, ***p* < 0.01. Abbreviations: *Cdh5*/VE-Cad: Cadherin 5 / Vascular-endothelial cadherin; *Icam2*: Intercellular adhesion molecule 2; *Klf2*: Kruppel-like factor 2; *Pecam1*/CD31: Platelet-endothelial cell adhesion molecule 1; *Vcam1*: Vascular cell adhesion molecule 1.


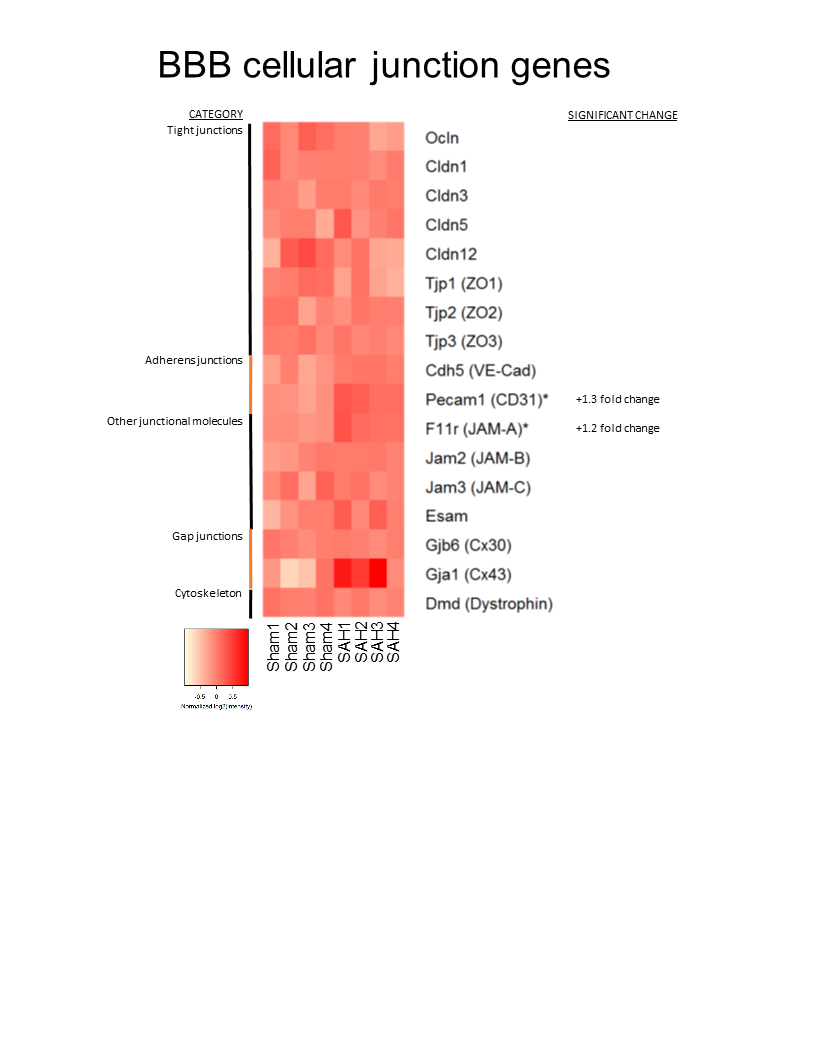


**Supplementary Figure S5. Expression changes of genes related to BBB intercellular junctions in CD45-CD31+ brain endothelial cells after SAH.** *corrected *p* < 0.05.


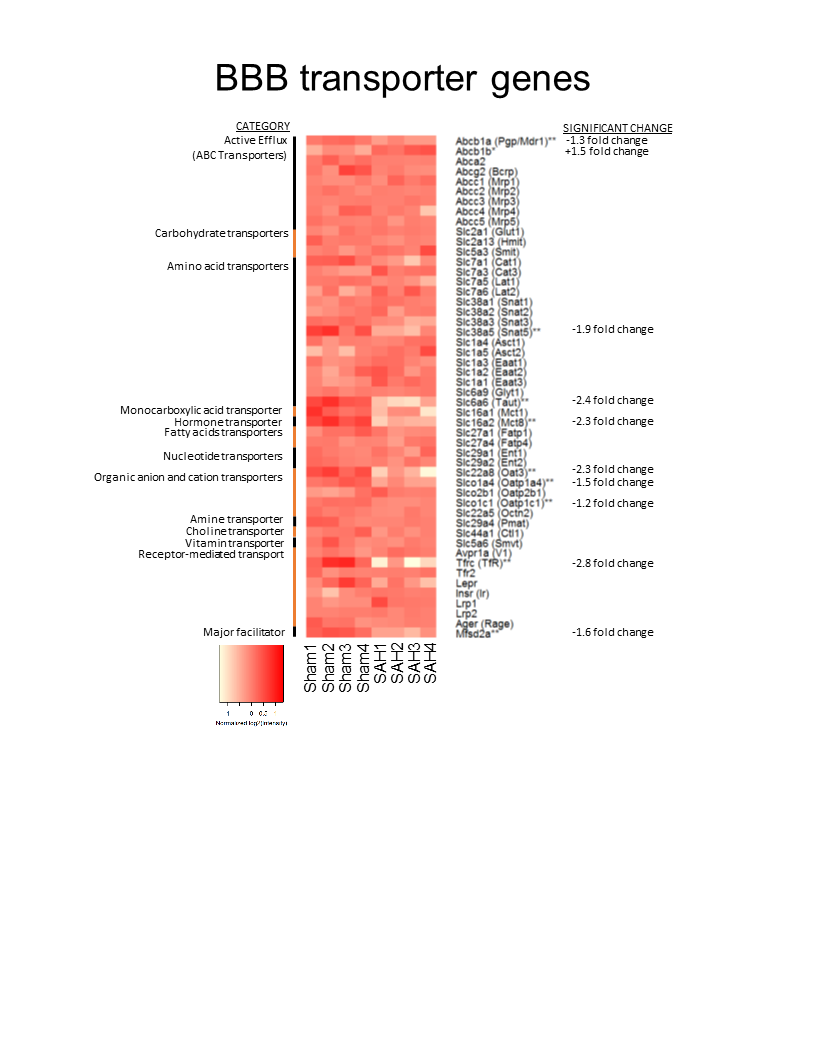


**Supplementary Figure S6. Expression changes of genes related to BBB transporters in CD45-CD31+ brain endothelial cells after SAH.** *corrected *p* < 0.05 (upregulated), **corrected *p* < 0.05 (downregulated).


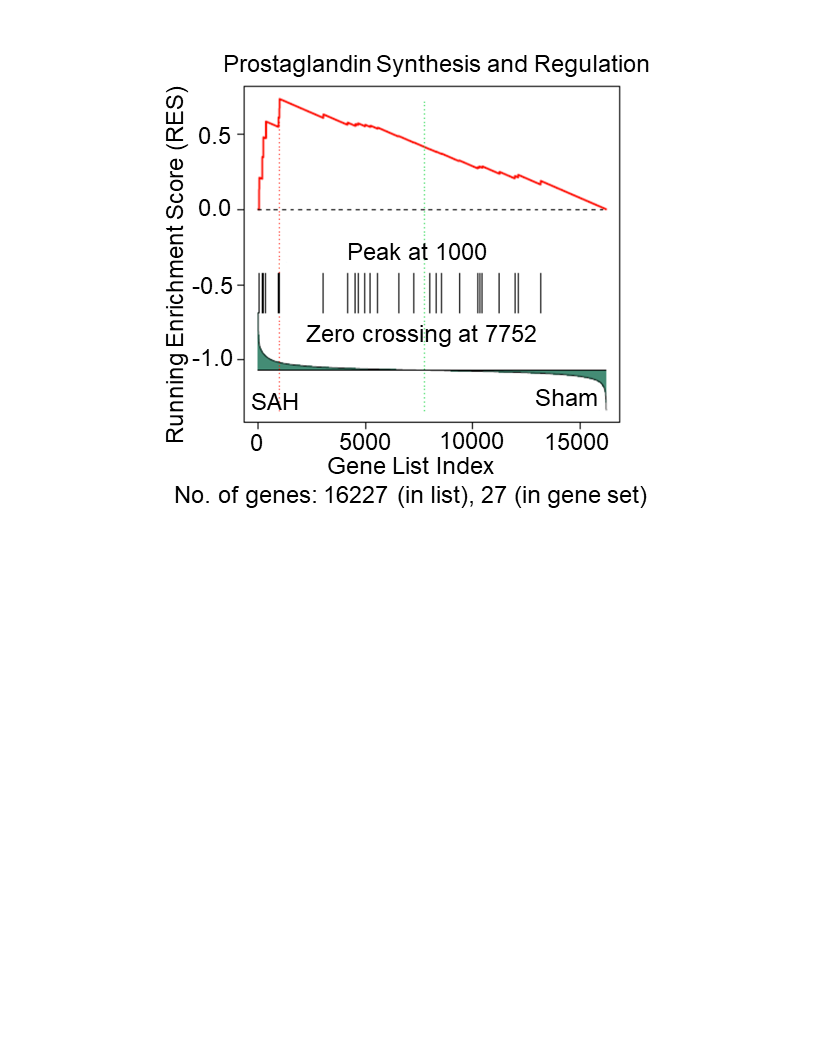


**Supplementary Figure S7. Gene set enrichment analysis (GSEA) showing upregulation of genes related to “prostaglandin synthesis and regulation” in CD45-CD31+ brain endothelial cells after SAH.**

**
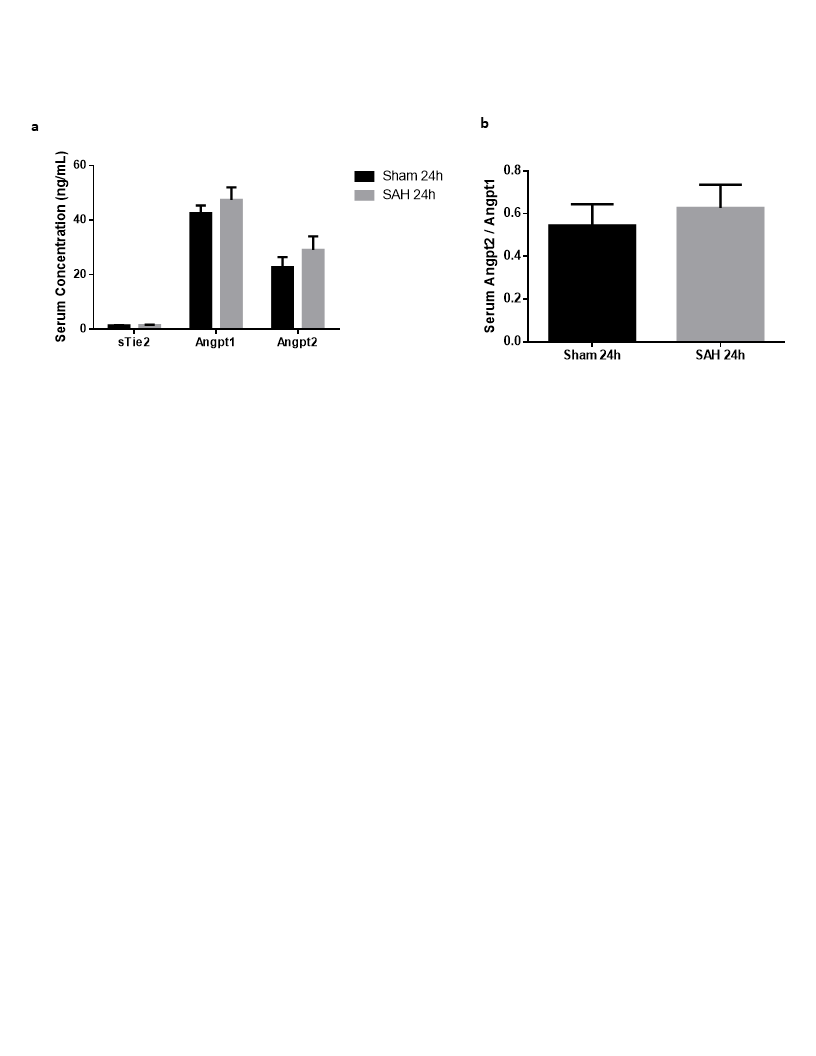
**

**Supplementary Figure S8. Expression of Angpt1, Angpt2, and sTie2 (soluble Tie2) in serum after experimental SAH.**  **(a-b)** Serum measurements of Angpt1, Angpt2, sTie2 and Angpt1/Angpt2 using enzyme-linked immunosorbent assay (ELISA) kits. *n* = 5. *t*-test with Holm-Sidak *post-hoc* correction **p* < 0.05. Abbreviations: Angpt1/2: Angiopoietin 1/2; ND: None detected; sTie2: Soluble tunica intima endothelial kinase 2; Tie2: Tunica intima endothelial kinase 2.


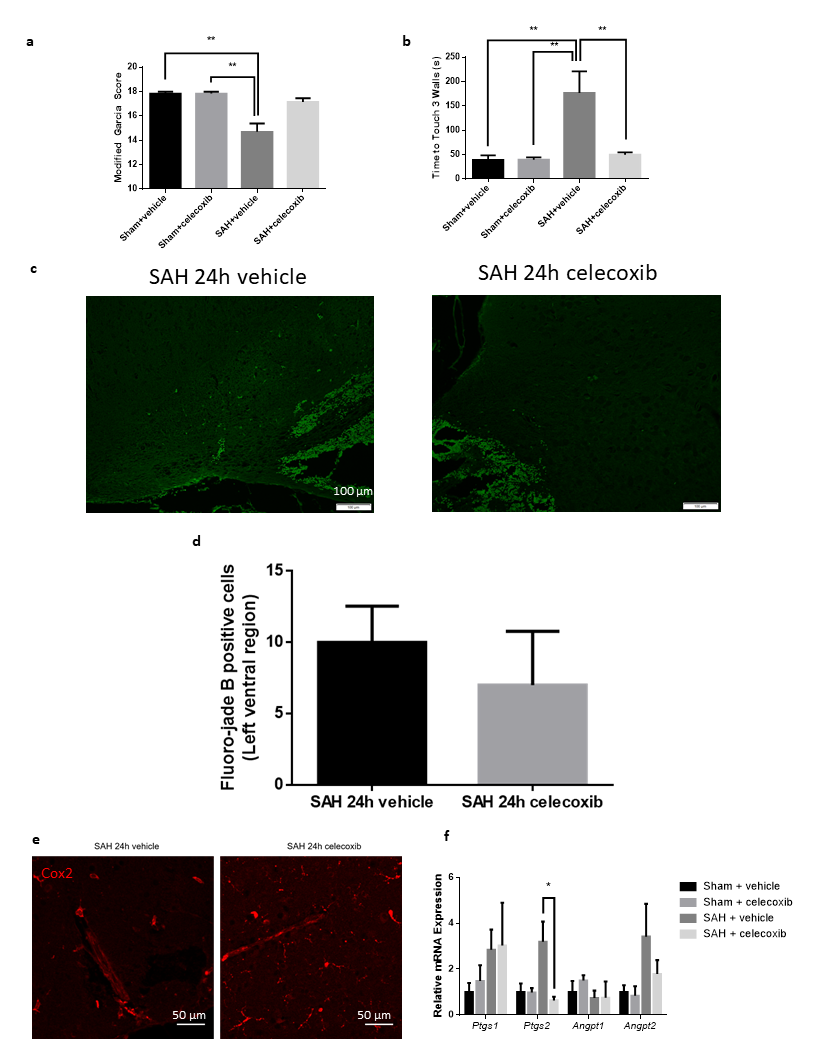


**Supplementary Figure S9. Celecoxib treatment in experimental SAH.** (**a**) Neurobehavioural assessments 24 h after SAH or sham procedure, treated with selective Cox2 inhibitor celecoxib or vehicle. Kruskal-Wallis test, **p < 0.01. (**b**) Activity level 24 h after SAH or sham procedure, treated with celecoxib or vehicle. One-way ANOVA with Holm-Sidak *post-hoc* correction, ***p* < 0.01. (**c-d)** Fluorescent microscopy of coronal brain slices stained with fluoro-jade B for degenerated neurons and quantification in left ventral region after SAH treated with celecoxib or vehicle. (**e**) Confocal microscopy of coronal brain slices after SAH, showing Cox2 expression in red. (**f**) mRNA expression of ptgs1, ptgs2, angpt1, and angpt2 in CD45-CD31+ BECs 24 h after SAH or sham procedure, treated with celecoxib or vehicle. *n* = 6-7 per group (a-d). *n* = 3-4 per group (e,f). Data presented as means ± SEM.


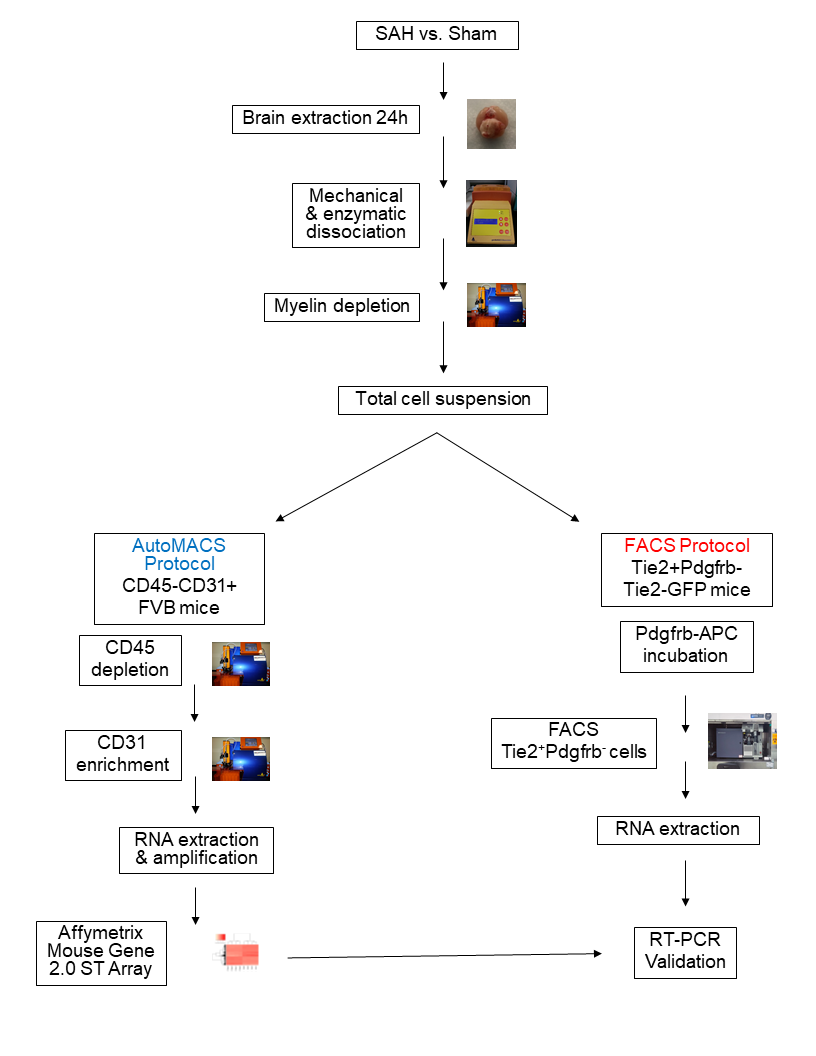


**Supplementary Figure S10. Workflow for isolating brain endothelial cells.**

**Supplementary Table S1**: Significantly upregulated and downregulated genes after experimental SAH

**Supplementary Table S2**: Gene-probe set data from microarray.

**Supplementary Table S3**: RT-PCR primers used.

**References**

1 Sabri, M. *et al.* Anterior circulation mouse model of subarachnoid hemorrhage. *Brain Res* **1295**, 179-185, doi:10.1016/j.brainres.2009.08.021 (2009).

2 Sherchan, P. *et al.* Minocycline improves functional outcomes, memory deficits, and histopathology after endovascular perforation-induced subarachnoid hemorrhage in rats. *J Neurotrauma* **28**, 2503-2512, doi:10.1089/neu.2011.1864 (2011).

3 Reagan-Shaw, S., Nihal, M. & Ahmad, N. Dose translation from animal to human studies revisited. *Faseb j* **22**, 659-661, doi:10.1096/fj.07-9574LSF (2008).

4 Stepita-Klauco, M. & Dolezalova, H. Cadaverine in the brain of axenic mice. *Nature* **252**, 158-159, doi:10.1038/252158a0 (1974).

5 Irizarry, R. A. *et al.* Summaries of Affymetrix GeneChip probe level data. *Nucleic Acids Res* **31**, e15, doi:10.1093/nar/gng015 (2003).

6 Benjamini, Y., Drai, D., Elmer, G., Kafkafi, N. & Golani, I. Controlling the false discovery rate in behavior genetics research. *Behav Brain Res* **125**, 279-284, doi:10.1016/s0166-4328(01)00297-2 (2001).

7 Subramanian, A. *et al.* Gene set enrichment analysis: a knowledge-based approach for interpreting genome-wide expression profiles. *Proc Natl Acad Sci U S A* **102**, 15545-15550, doi:10.1073/pnas.0506580102 (2005).
