# Supplementary material for: Gene expression profiling of brain endothelial cells after experimental subarachnoid haemorrhage
Source: Sci Rep. 2021 Apr 9;11:7818. doi: 10.1038/s41598-021-87301-z (PMC8035152; doi:10.1038/s41598-021-87301-z)
Supplement: Supplementary file 4 — Supplementary Table S3. [file 41598_2021_87301_MOESM4_ESM.docx]

Supplementary Table S3. Forward and reverse primer sequences used for RT-PCR.

| **Gene name** | **Accession Number** | **Forward Primer (5' - 3')** | **Reverse Primer (5' - 3')** |
| --- | --- | --- | --- |
| *Actb* (β-actin) | NM_007393.5 | AAC CGT GAA AAG ATG ACC CAG AT | CAC AGC CTG GAT GGC TAC GTA |
| *Angpt1* | NM_009640.4 | GGC CAC CAT GCT TGA GAT AG | CAA GTC GGG ATG TTT GAT TTA |
| *Angpt2* | NM_007426.4 | GCA GCT TCT CCA ACA TTC TA | CCT CCA TGT CCA GAA CTT TC |
| *Cdh5* (VE-Cadherin) | NM_009868.4 | TGC CCA CCA TCG CCA AAA GAG AGA C | CTG GCG GTT CAC GTT GGA CTT G |
| *Hif1a* | NM_001313919.1 | TCA GTT GCC ACT TCC CCA CAA | AGA CCA CCG GCA TCC AGA AGT T |
| *Kdr/Vegfr2* | NM_010612.2 | ACC GGG ACG TCG ACA TAG | CAC TGA CAG AGG CGA TGA A |
| *Klf2* | NM_008452.2 | CTG CGG CAA GAC CTA CAC CAA | CGC ATC CTT CCC AGT TGC AAT GAT A |
| *Luciferase* | U47123.2 | ACT CCT CTG GAT CTA CTG GTC | GTA ATC CTG AAG GCT CCT CA |
| *Mfsd2a* | NM_029662.2 | GGC TGC GCA CTG GGA TTC T | CCA GGC TCG GCC CAC AAA |
| *Pecam1* (CD31) | NM_008816.3 | TCA GAA CCC ATC AGG AGT GAA TAC G | TGC TTG GAG GTG GCT ACA ATC |
| *Ptgs1/Cox1* | NM_008969.4 | GAC CAC TCG CCT CAT CCT TAT A | TCA AAC TTG AGC TGC AGG AAA TA |
| *Ptgs2/Cox2* | NM_011198.4 | CCT TCC TGC GAA GTT TAA T | GGT GGA CTG TCA ATC AAA TAT |
| *Rela* (p65) | NM_009045.4 | TGCG GTG GGG ATG AGA TCT T | CAG CCT GGT CCC GTG AAA TA |
| *Tek/Tie2* | NM_013690.3 | GTT CGA GGA CAG GCT ATA A | TGT CCA CGG TCA TAG TTA AA |
| *Vcam1* | NM_011693.3 | GGG AAG CTG GAA CGA AGT AT | GGG GCC ACT GAA TTG AAT CTC |
